# Effectiveness of resilience-based interventions to promote mental well-being among secondary school children: a systematic review

**DOI:** 10.3389/fpsyt.2026.1642660

**Published:** 2026-03-05

**Authors:** Andrea Utz Matus de la Parra, Marion Gibbon, Dean M. Thompson

**Affiliations:** 1 Department of Applied Health Sciences, School of Health Sciences, College of Medicine and Health, University of Birmingham, Birmingham, United Kingdom; 2 Public Health Division, Birmingham City Council, Birmingham, United Kingdom

**Keywords:** adolescent, child, mental health, resilience, school, systematic review

## Abstract

**Background:**

Mental health conditions are becoming increasingly prevalent among school children. Well-being and resilience are vital for preventing and mitigating the severity of mental health issues. Equipping children with effective coping mechanisms and protective behaviours enhances their resilience to manage challenging life circumstances, leading to improved mental health. While evidence supports the effectiveness of resilience-based interventions in reducing mental health problems in adolescents, it is unclear which components enhance resilience most effectively and how follow-up duration influences outcomes. Furthermore, little is known about the facilitators and barriers impacting successful implementation of these interventions in school settings. The aim of this review was to identify effective resilience-based intervention components, facilitators and barriers, and outcome variation by follow-up period.

**Methods:**

A systematic review and narrative synthesis was carried out, with five electronic databases (EMBASE, MEDLINE, PsycINFO, CINAHL, and CENTRAL) searched to identify trials on resilience-based interventions that reported a measure of mental health problems or resilience in children or adolescents aged 11 to 19 attending secondary education.

**Results:**

A total of 34 trials were included from 3,419 records. Across trials, twelve resilience-based interventions were found to be effective compared to a control or alternative intervention for 5 of 9 outcomes: depressive symptoms, anxiety symptoms, resilience, emotional symptoms, and externalising problems. Interventions employing a multicomponent approach demonstrated significant effects in six of twelve trials showing positive results, particularly those combining social skills training, cognitive behavioural therapy (CBT) and positive psychology. Group-based interventions, especially those incorporating peer collaboration and social learning, appeared to be more effective. At short-term follow-up, ten studies reported significant effects across at least one outcome. These included small effects for anxiety, resilience, and externalising problems (k = 4), moderate effects for depressive symptoms and resilience (k = 3), and large effects for resilience and depressive symptoms (k = 2). Notably, one study yielded three significant outcomes, namely depressive symptoms, externalising problems, and resilience. At long-term follow-up, small but significant effects were reported in two studies (k = 2) for depressive and emotional symptoms.

**Conclusion:**

The findings provide evidence that resilience-based interventions can improve mental health outcomes for school children, including reducing depressive and anxiety symptoms, externalising problems, and increasing resilience. Future research should focus on addressing barriers like student engagement and implementation fidelity.

## Introduction

Mental health conditions frequently emerge through a mix of negative thinking patterns, maladaptive behaviours, distressing emotions, and challenging interpersonal relationships, with their impact varying from mild to severe functional impairment (1). There are several factors that contribute to the risk of poor mental well-being in childhood, including social, family, psychological and genetic factors (2). Nonetheless, this risk during the early years is primarily associated with social adversity, such as socioeconomic hardship, racial discrimination, abuse and neglect (3), intergenerational trauma, bullying, and community violence (4). Globally, higher rates of mental health issues are observed in young people from lower socio-economic backgrounds, minority ethnic groups (5), and rural or isolated areas (6).

The peak onset of most mental disorders occurs during the transition from childhood to young adulthood, with up to one in five individuals facing clinically significant mental health issues before the age of 25 (5). It is estimated that 50% of mental health conditions begin around the age of 14, but most cases are neither detected nor treated (7). Although many of these conditions can be effectively treated, the quality of care frequently falls short (8), and stigma remains an obstacle for children trying to seek help (9). Furthermore, resources for mental health are unfairly distributed across communities, being the adult mental health services typically prioritised over services for children (10), which leads to less available care for this group.

The most recent Global Burden of Disease (GBD) study reported that in 2019, mental disorders were responsible for 21.5 million years of healthy life lost due to disability (YLDs) among individuals aged 0–19 worldwide. Depression, anxiety, and behavioural disorders rank among the top causes of illness and disability in adolescents (11).

A significant number of children and adolescents are affected by mental health conditions, with rates reaching alarming levels (12). Globally, one billion people are affected by some form of mental disorder, as reported by the latest United Nations data, which includes over one in seven adolescents (13). The picture in the EU indicates that approximately 13% of children and young people under 19 experience a mental health condition (UNICEF, 2024) (14). This issue is also evident in the UK, where the rate of probable mental disorders in children aged 8 to 16 rose from 17.1% to 20.3% between 2020 and 2023 (NHS) (15). Mental health disorders represent the single largest economic burden globally. This is evident in the rising cost of treating depression and anxiety, which now exceeds US$ one trillion per year (16).

In response to the growing prevalence and substantial economic burden of mental health conditions, there has been a shift towards preventive approaches that prioritise the promotion and maintenance of wellbeing (13). Secondary schools have been identified as a critical setting for such approaches, as they provide universal, non-stigmatising access to adolescents during a developmental period when many mental health difficulties first emerge. Early school-based interventions are increasingly recognised as cost-effective strategies that reduce future demand for healthcare services, reinforcing the principle that prevention is more sustainable than cure (17). By embedding interventions within the school environment, it is possible to reach entire student populations and strengthen resilience and wellbeing at a population level, prior to the escalation of mental health needs (13, 17).

### Resilience

In recent years, resilience has emerged as an important outcome for promoting positive mental health in young people (18). Resilience is defined as an interactive process that enables positive adaptation in the face of significant adversity or threat (19). It encompasses the accumulation and application of skills, abilities, knowledge, and insights that facilitate navigation and the successful overcoming of life’s challenges (20, 21). Rather than being a fixed trait, resilience is a capacity that exists within everyone and is often most evident when it is most needed (22). At the core of this process lies the dynamic interplay between internal and external protective factors (18).

Drawing from this understanding, resilience can be conceptualised as a process that fosters positive development and competence despite encountering adverse life circumstances by leveraging these protective factors (18).

The internal protective factors include personal characteristics or strengths, while external protective factors encompass elements of a child’s environment, such as family, social, and community support (23). When effectively combined, these protective factors enable individuals to adapt, thrive and overcome disadvantages (24). However, a systematic review by Fritz (25) emphasises that these protective factors do not operate in isolation but interact as part of a dynamic system. Evidence suggests that resilience is better understood through the interplay of multiple factors and higher-order adaptive processes, such as positive appraisal styles and flexible regulation of stress responses, rather than as the result of static traits alone (25).

The key protective factors involved in the resilience process include spirituality, social support networks, interpersonal relationships, family support, self-efficacy, physical activity, coping and perseverance, self-regulation, competence, empathy, self-esteem, and social skills (26). Therefore, resilience-based interventions aim to target these protective factors and strengthen them as a recommended approach to promote mental well-being.

Childhood is particularly conducive to fostering resilience (27), since it is a critical stage for the onset of mental disorders and interventions during this time have been encouraged by the World Health Organization (WHO) to equip young people with coping skills to handle stressful life events (28). Additionally, the school setting represents a practical and influential context for the delivery of mental health promotion interventions, offering sustained access to young people and opportunities to build resilience within everyday educational practice (29).

### Resilience theory and models

According to Grotberg, resilience is not solely a response to adversity but can be nurtured proactively, preparing individuals for inevitable life challenges (30). Masten and Obradović identify nine core adaptive systems, including emotional, cognitive, relational, and sociocultural systems, that play a foundational role in supporting resilience (31). When these systems remain intact and functioning well, resilience is common. However, when disrupted, targeted interventions may be necessary to prevent developmental risks (32).

Cove et al. classified existing models of resilience into three categories; compensatory (neutralising risks), challenge (framing stressors as growth opportunities), and protective factor models (modifying responses to risk), which can offer different yet complementary approaches to fostering resilience (33). These models suggest that prevention programs should not focus merely on reducing risks but also on strengthening the protective systems around children (32).

### Resilience-based interventions

Resilience-based interventions are diverse and can differ in different aspects, such as the mode of delivery, ranging from structured curriculum-based lessons to broader capacity-building approaches to enhance protective factors. These interventions also vary in terms of lesson frequency, overall length of the program, facilitator, and whether the format is in-person or online (18).

Typically implemented within school settings, these interventions often follow a universal approach, aiming to support entire student populations rather than focusing on individuals identified as at risk or experiencing mental health difficulties (34).

A systematic review by Dray (18) evaluated the effectiveness of resilience-based interventions aimed at improving the mental health of children and adolescents between 5 to 18 years in school settings. Quantitative synthesis across 49 trials demonstrated small but significant pooled effects in favour of resilience-based interventions for several outcomes, including depressive symptoms (SMD = -0.08, 95% CI [-0.14 to -0.01]), internalising problems (SMD = -0.21, 95% CI [-0.36 to -0.06]), externalising problems (SMD = -0.18, 95% CI [-0.34 to -0.01]), and general psychological distress (SMD = -0.11, 95% CI [-0.21 to -0.01]) (18). While these interventions were found to have positive effects in reducing depressive and anxiety symptoms, substantial heterogeneity, variability in intervention components and follow-up duration, and a high overall risk of bias -largely due to lack of blinding and reliance on self-report measures- limited conclusions regarding long-term effectiveness and the most effective intervention characteristics. These limitations underscore the need for updated evidence by incorporating more recent studies that may offer improved methodological quality.

A more recent systematic review and meta-analysis by Pinto et al. (27) synthesised evidence from 17 randomised controlled trials employing group-based psychotherapeutic approaches commonly grounded in cognitive behavioural frameworks. The review reported a moderate overall effect on resilience outcomes immediately post-intervention (SMD = 0.48, 95% CI: [0.15-0.81], *p* = 0.0077) (27). Subgroup analyses indicated that significant effects were observed primarily among adolescents, whereas evidence for children was less consistent. The authors also highlighted substantial heterogeneity across programmes, limited follow-up beyond six months and a generally high risk of bias. This underscores ongoing uncertainties regarding the sustainability of effects and the most effective intervention components. Llistosella’s (26) systematic review suggested that interventions targeting resilience in early adolescents within the school setting were effective in the short-term but provided limited evidence of sustained impact on mental health outcomes. The pooled analysis showed a moderate overall increase in resilience following intervention (SMD = 0.58, 95% CI [0.29-0.87]), with substantially larger effects observed at short-term follow-up (≤ 8 weeks) (SMD = 1.54, 95% CI [0.61-2.47]). Effects were significant primarily among at-risk adolescents (SMD = 1.28, 95% CI [0.53-2.02]) and for multicomponent interventions (SMD = 1.45, 95% CI [0.11-2.80]), while no meaningful effects were observed in general population samples or beyond short follow-up periods (26). Furthermore, considerable heterogeneity across studies and variation in intervention components and outcome measures limited conclusions regarding which specific elements were most effective at enhancing resilience.

Fenwick-Smith (35) conducted a systematic review of interventions targeting school children aged 5–12 years, reporting consistent short-term improvements in resilience-related outcomes, including coping and emotional regulation (35). However, the review did not include a meta-analysis or report pooled effect sizes, due to substantial heterogeneity in study designs and outcome measures. Moreover, this review also noted a lack of evidence for long-term effects and offered limited insights into which elements contributed most to intervention success. While these reviews support the short-term effectiveness of resilience-based interventions in school settings, they have not fully explored the components that underpin and sustain these outcomes.

Similarly, Higgen (36) reviewed 17 studies on universal mental health interventions for students in challenging environments, finding resilience improvements in 15 through classroom-based programs. However, Higgen underscores the need for high-quality research to evaluate the effectiveness of resources linked to social and physical environments (36).

While previous reviews have primarily focused on the overall effectiveness of resilience-based interventions, none have systematically examined facilitators and barriers to implementation, and they have provided limited insight into which intervention components and delivery characteristics underpin and sustain outcomes. This review addresses this gap by synthesising evidence on intervention components, delivery formats, and implementation facilitators and barriers, alongside follow-up duration and mental health and wellbeing outcomes, to clarify how and under what conditions resilience-based interventions in secondary school settings are most effective. We aim to address the following research questions:

1. How effective are resilience-based interventions in improving mental health outcomes among secondary school children?
2. What are the key components of resilience-based interventions delivered in secondary school settings?
3. What facilitators and barriers influence the implementation of resilience-based interventions in secondary schools?
4. How does the impact of resilience-based interventions vary across different follow-up timepoints?

## Methods

This systematic review was conducted and reported in accordance with PRISMA guidance (see **Supplementary Material 1** for the PRISMA checklist) (37) and was structured using the PICO framework (Population, Intervention, Comparison and Outcome) (38).

### Protocol registration

A systematic review protocol was written with reference to PRISMA-P guidelines (39) and registered on the International Prospective Register of Systematic Reviews (PROSPERO) on 17 June 2024: CRD42024558070.

### Eligibility criteria

#### Inclusion criteria

Only randomised controlled trials (RCTs) and cluster randomised controlled trials were eligible. The review included children and adolescents 11 to 19 years of age enrolled in secondary school or equivalent post-compulsory education. Studies were included if one or more intervention was resilience-based and comprised at least three internal protective factors, such as coping, emotional regulation, and self-efficacy (23). This criterion was applied to ensure conceptual consistency with models of resilience, which view resilience as an evolving process resulting from the interaction of various individual-level protective factors, rather than as a single skill or trait (40). Studies were included only if resilience interventions were compared with a control group (no intervention, usual practice, or another intervention). Studies were included only if they measured mental health outcomes including depressive symptoms, anxiety, internalising/externalising problems, conduct problems, or general psychological distress. Additionally, studies were included only if the intervention took place in a secondary school setting or equivalent educational context. Interventions were included regardless of delivery mode, activity type, format, duration or length of follow-up. If studies included content related to both resilience and non-resilience elements, details of each were extracted separately. The effect of the resilience intervention component was isolated and reported in this review. Only studies from the last 11 years were included, between 2014 and 2025 November. Given the evolving nature of school-based mental health strategies and resilience-focused interventions, this ensured a focus on the most recent evidence contextualised within current digital advancements (41). Studies were included only if they were published in English or Spanish.

#### Exclusion criteria

Studies were excluded if they used non-randomised designs, including controlled before-and-after studies, cohort studies, case-control studies, cross-sectional studies, case series, or case reports. Studies were further excluded if they involved participants with pre-existing diagnosed mental health conditions or developmental disabilities, were conducted outside the target age range of 11–19 years, involved interventions delivered outside secondary school settings, or were published in languages other than English or Spanish.

### Information sources

Five electronic databases were searched on 26^th^ November 2025 to identify relevant studies: EMBASE (via OVID); MEDLINE (via OVID); PsycINFO (via OVID); CINAHL (Cumulative Index to Nursing and Allied Health Literature); CENTRAL (Cochrane Central Register of Controlled Trials, The Cochrane Library). Hand searches of the reference lists of the included studies were performed.

### Search strategy

The search strategy included terms related to intervention, population, and outcome. The search terms were developed from an existing review on the same topic (42). The search strategy was adjusted where necessary for searching individual electronic databases (see **Supplementary Material 2**).

### Study selection

All retrieved studies from the databases were exported to EndNote (43), and search results were deduplicated automatically and then manually. The titles and abstracts for inclusion in the review were screened by three independent reviewers according to the predefined inclusion criteria. The first reviewer (AU) screened 90% of the titles and abstracts, while the second (MG) and third (DT) reviewers each screened 5%. Full-text articles meeting the inclusion criteria were assessed independently by all three reviewers.

### Data extraction

Data collection was performed according to a pre-piloted data extraction, which was uploaded to the Covidence (44) software for remote collaborative data extraction.

Two independent reviewers (AU, DT) extracted and included data on:

(1) Study information: Title, author, institution, study country, sponsorship source, year of publication, and publication language; (2) Method: Study design; (3) Participant characteristics: Sample size, use of power calculation, age group, gender, and setting; (4) Intervention characteristics: Mode of delivery, brief description of the programme, activity type and components, frequency, facilitators and barriers, and duration of the intervention; (5) Outcomes characteristics: outcome reported, time points, scale/tool used, unit of measurement, direction of effect, and total length of follow-up; (6) Results: measure of effect; Mean difference (MD) and Standardised Mean Difference (SMD).

### Risk of bias assessment

The Cochrane Risk of Bias Tool (45) was used to assess the individual risk of bias. One reviewer (AU) performed a critical appraisal of the risk of bias against random sequence generation, allocation concealment, blinding of participants and personnel, blinding of outcome assessment, incomplete outcome data, and selective reporting. Each study was assigned an overall risk of bias according to three categories (46): low, some concerns, or high risk. The risk of bias assessment was piloted before initiating the formal process in case any amendments were required.

### Data synthesis and analysis

The findings from the included studies were organised by outcome using narrative synthesis. This was conducted in accordance with Popay and colleagues’ process (47), comprising a preliminary synthesis, exploration of the relationship within and between studies, and assessment of robustness in relation to evidence synthesis. A tabulated format with narrative text is presented, including the relevant characteristics from each study. A scoping search indicated a narrative synthesis was appropriate given the prevalence of a range of sources of heterogeneity. Information on implementation barriers and facilitators was identified from narrative descriptions within the included studies. Relevant text was extracted and synthesised narratively, with implementation factors coded into predefined categories informed by the Consolidated Framework for Implementation Research (CFIR) (48). The data extracted from studies reporting follow-up assessments were categorised into two groups: short-term (<6 months) and long-term (>6 months) effects. For studies reporting more than one follow-up assessment, the most recent endpoint was selected for data utilisation and reporting. This approach allowed the capture of the longest available effects of the intervention. As all studies reported continuous outcomes, the mean difference (MD) was presented where measures of outcomes were consistent across studies. A standardised mean difference (SMD) was used if different measures were used to report a comparable outcome. A threshold of 0.05 was considered statistically significant.

## Results

### Study selection

**Figure 1:**
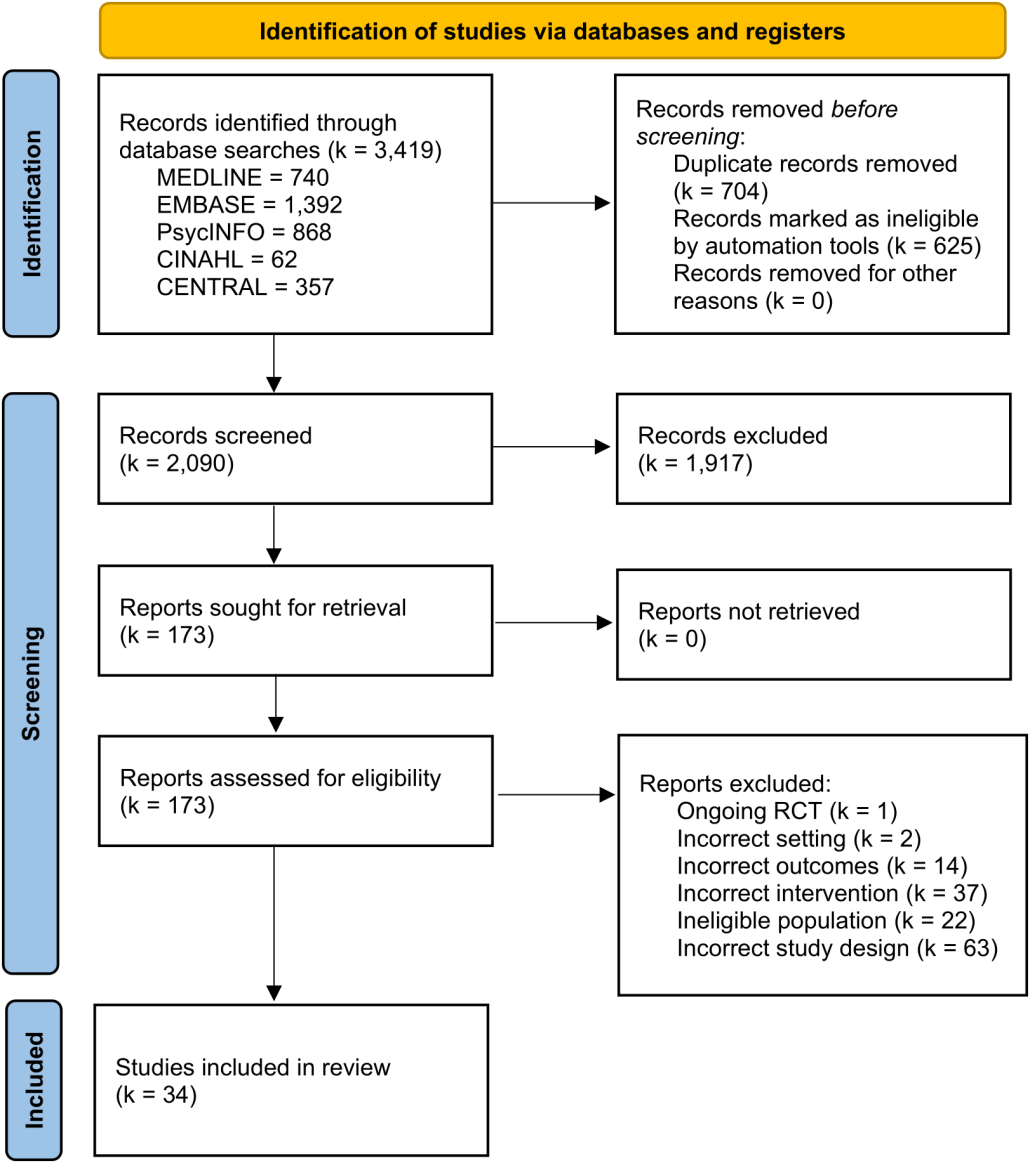
PRISMA flow diagram showing the selection process of records (37).

The literature search identified a total of 3,419 records, with no additional sources identified through the bibliographic examination of the included papers. After manually removing duplicates and records marked as ineligible by automation tools, 2,090 studies remained. Following the title and abstract screening process, 1,917 records did not meet the inclusion criteria and were excluded. The full-text screening of 173 records was assessed for eligibility, which resulted in 139 records excluded. Thirty-four studies were included. The complete selection process is detailed in the PRISMA flow diagram (37) (see **Figure 1**).

The reasons for exclusion at the stage of full-text screening were predominantly related to ineligible study design. See **Supplementary Material 3** for studies excluded after full-text screening with justification.

### Development of preliminary synthesis and relationship exploration between studies

#### Study characteristics

The characteristics of the studies included (49–82) in the review are presented in **Supplementary Table S1**. The study designs employed were Cluster RCT (k=19), and RCT (k=15). All 34 studies displayed variability with respect to the population under investigation; the age range of adolescent participants spanned from 11 to 18 years. One study reported that only female participants were included (Girls First Resilience Curriculum) (63).

**Figure 2:**
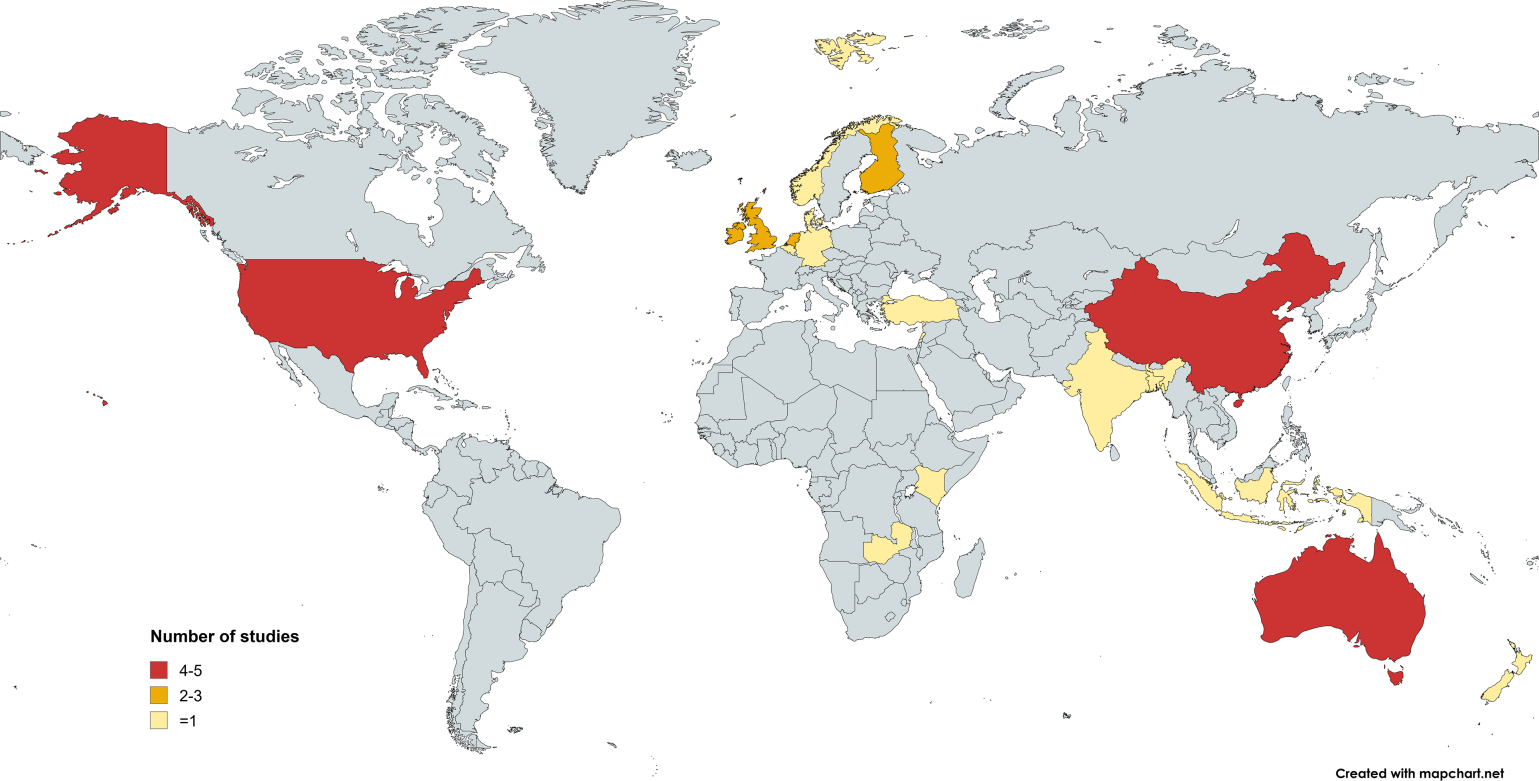
Country/region from included studies.

The study settings encompassed a diverse range of countries (see **Figure 2**), including Australia (k=5), China (k=5), USA (k=4) the UK, Ireland, Finland, and the Netherlands (k=2); Germany, Norway, Denmark, Belgium, New Zealand, Turkey, India, Lebanon, Indonesia, Bangladesh, Kenya, and Zambia (k=1). The sample sizes indicated considerable variation across studies, ranging from small groups of 60 participants to large-scale studies involving over 8,000 students.

### Resilience-based intervention description

Most of the interventions were conducted within the school setting, employing a group-based format (k=25). A minority of studies were conducted online (k=5), employed a combination of face-to-face and online delivery (k=3), or used an app-based format (k=1). The principal components of the interventions were based on the principles of resilience and coping skills (k=9), cognitive-behavioural therapy (k=8), mindfulness (k=6), positive psychology (k=4), physical activity (k=3), social and emotional learning (k=2), acceptance and commitment therapy, and occupational therapy (k=2). The sessions most frequently involved activities of group discussion (k=13), storytelling (k=3), Role-playing, worksheets (k=2), infographics, and self-monitoring of emotions (k=1).

Regarding the duration of the intervention, most studies lasted between eight to twelve weeks (k=15). A few studies had a shorter duration of four to six weeks (k=7), while others extended beyond 12 weeks (k=8). Additionally, some studies employed a single-session intervention (k=4). Most studies reported interventions with a frequency of one contact per week, with a range of follow-up periods between two weeks and over 12 months follow-up. One study reported the longest follow-up period of three years. For categorisation purposes, studies were divided based on their follow-up duration into short-term (<6 months) and long-term (>6 months) outcome effects. Specifically, 27 studies were classified as short-term, while 7 studies were categorised as long-term.

### Outcome domains

Nine outcome domains were identified. Depressive symptoms were the most frequently reported outcome (k=19), followed by anxiety symptoms (k=14), resilience (k=13), internalising problems, externalising problems, emotional distress, and conduct problems (k=2). Other outcomes included general psychological distress, and academic buoyancy (k=1).

### Effectiveness

#### Short-term effects

##### Depressive symptoms

The majority of studies evaluating the effectiveness of resilience-based intervention on depression reported statistically non-significant effects (k=12). However, three studies reported significant positive effects in favour of resilience interventions. Among the included studies, one involved a physical activity component through circuit training sessions (49), one focused exclusively on positive psychology (75), and another combined positive psychology with acceptance and commitment therapy (51). In particular, the first study yielded a large effect size, while the second and third studies demonstrated moderate effect sizes in reducing depressive symptoms.

##### Anxiety symptoms

Studies reporting a measure of anxiety symptoms demonstrated predominantly non-statistically significant effects (k=10). Nevertheless, three studies reported a small yet statistically significant positive effect on reducing anxiety symptoms. One intervention targeted girls using a positive psychology and emotional intelligence approach (63), another employed a combined gender approach through a smartphone-based self-help app using Cognitive behavioural therapy (71), and a third focused on strengthening coping and emotion regulation skills through structured psychoeducation and skills-based activities (73).

##### Resilience

For the resilience outcome domain, most studies reported non-statistically significant effects (k=8), except for five studies that showed varying magnitudes of effect sizes. The SPARK program yielded a large effect size (56), while the Sports-Based Youth Development Programme showed a small effect size (57), but both were statistically significant in improving resilience. Similarly, ACTi and Promotion Resilience demonstrated small but significant effects on resilience (53), the STRONG programme showed a moderate effect size (75), and the physical activity programme produced a large effect size in resilience outcomes (82). The SPARK programme was based on a Social and Emotional learning approach, while the Sports-Based Youth Development Programme focused on positive youth development principles through the practice of sports as an opportunity to socialise and develop social skills (56, 57). ACTi and Promotion Resilience were grounded in cognitive and acceptance-based resilience training delivered through structured group sessions (53), the STRONG programme focused on positive psychology-based resilience skill development (75), and the physical activity programme emphasised resilience enhancement through structured exercise and team-based activities (82).An overview of the short-term effectiveness results is summarised in **Supplementary Table S2**.

#### Long-term effects

Most of the seven interventions with long-term follow-up data demonstrated non-statistically significant results in terms of their effectiveness on the identified outcome domains, except for two studies.

##### Emotional symptoms

One study reported a small but significant effect on emotional symptoms favouring the intervention, after 1-year follow-up. The intervention involved a combination of self-management therapy and cognitive behavioural therapy, with an emphasis on building social competency.

##### Depressive symptoms

One study reported a small but significant reduction in depressive symptoms after a 20-month follow-up period. The intervention included a combination of rational emotive therapy and social learning theory, emphasising the development of interpersonal problem-solving skills with social reinforcement.

A summary of the long-term effectiveness of interventions is presented in **Supplementary Table S3**.

### Facilitators and barriers

Ten implementation themes comprising a set of facilitators and barriers were identified across the included studies and mapped to the Consolidated Framework for Implementation Research (CFIR) (48) (See **Table 1**). These implementation factors were primarily reported by teachers and intervention facilitators and were identified through author-reported observations.

**Table 1:** Facilitators and barriers to resilience-based interventions according to the consolidated framework for implementation research (CFIR).

CFIR domain	Implementation theme	Description	n
Intervention characteristics	Standardised format	There is consistency in the delivery of the intervention across schools, using structured manual and worksheets.	13
	Multi-component approach	Using a combined approach is advantageous for a more comprehensive and engaging intervention.	12
Inner setting	School-based setting	Easily accessible for many students, which makes it convenient and far-reaching.	34
	Teacher-led	Using teachers as facilitators to deliver the intervention is cost-effective and accessible to schools.	12
	Integration into school curriculum	It is more feasible to sustain the programme as the intervention is implemented during regular school hours.	10
	Limited resources	Implementing the intervention in a low-resource setting may limit the availability of necessary resources and support for teachers and facilitators.	5
Outer setting	Cultural considerations	The intervention might not be directly applicable to other contexts, particularly those with different cultural norms or educational systems.	12
Characteristics of individuals	Lack of engagement	Students present a lack of motivation or find the activities challenging to fully engage with the program.	5
Implementation process	Attrition	Large number of students lost to follow-up due to competing activities and lack of engagement.	19
	Implementation fidelity	The program is not being delivered consistently according to its intended plan or protocol.	4

Within the Intervention characteristics domain, commonly reported facilitators included standardised formats (k=13) and multi-component approaches (k=12). Within the Inner setting domain, school-based delivery was the most frequently reported facilitator (k=34), followed by teacher-led implementation (k=12) and integration into the school curriculum (k=10), while limited resources were reported as a barrier (k=5). Outer setting barriers included cultural considerations (k=12). Within the Characteristics of individuals domain, lack of student engagement was reported as a barrier (k=5). Process-related challenges included attrition (k=19) and implementation fidelity (k=4). Where reported, studies described implementation strategies such as adaptation of materials and additional facilitator training.

### Assessment of the robustness of included studies

**Figure 3:**
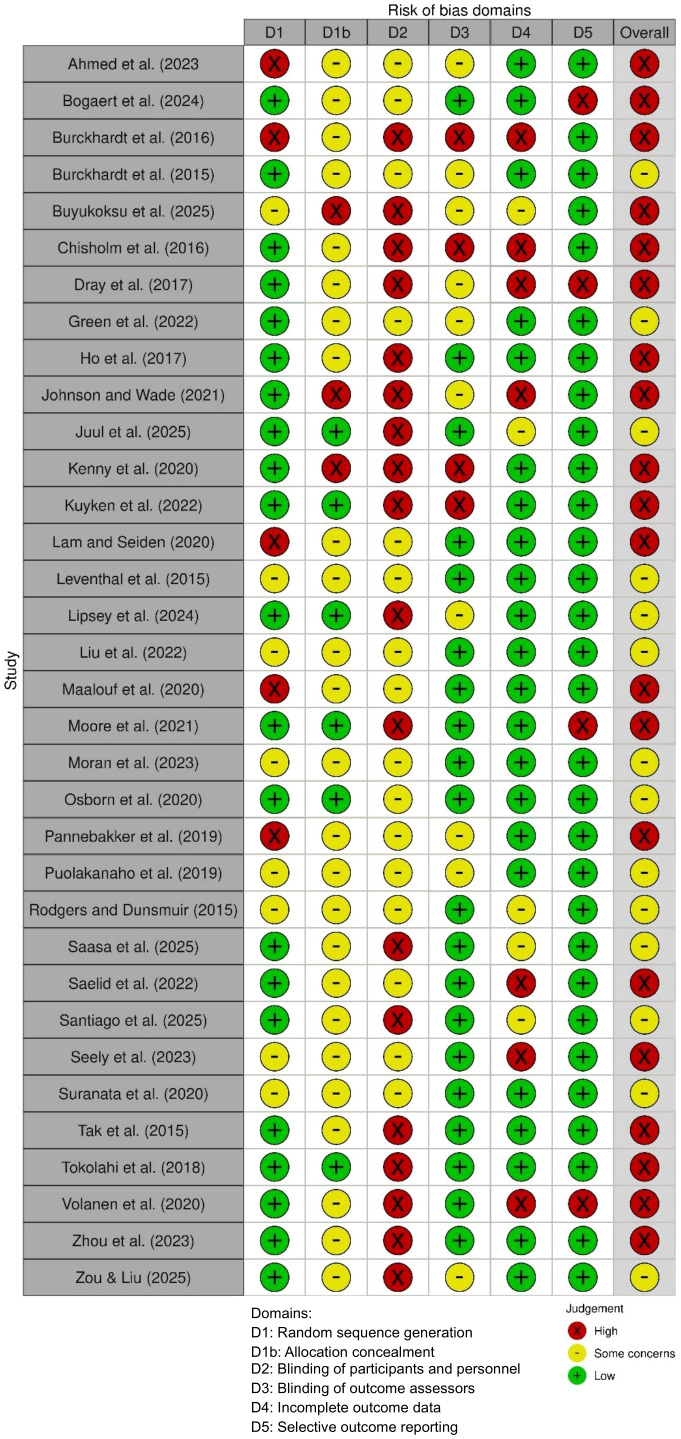
Risk of bias of included studies (46).

The risk of bias assessment of thirty-four included RCTs and cluster RCTs demonstrated a range of methodological sources of bias (46) (See **Figure 3**), most frequently related to allocation concealment and blinding of participants and personnel. Nineteen studies were deemed to have a high overall risk of bias, with an additional nine studies classified as moderate risk. No study was rated as low risk of bias. Studies with a moderate risk of bias were primarily attributed to inadequate reporting of allocation concealment.

**Figure 4:**
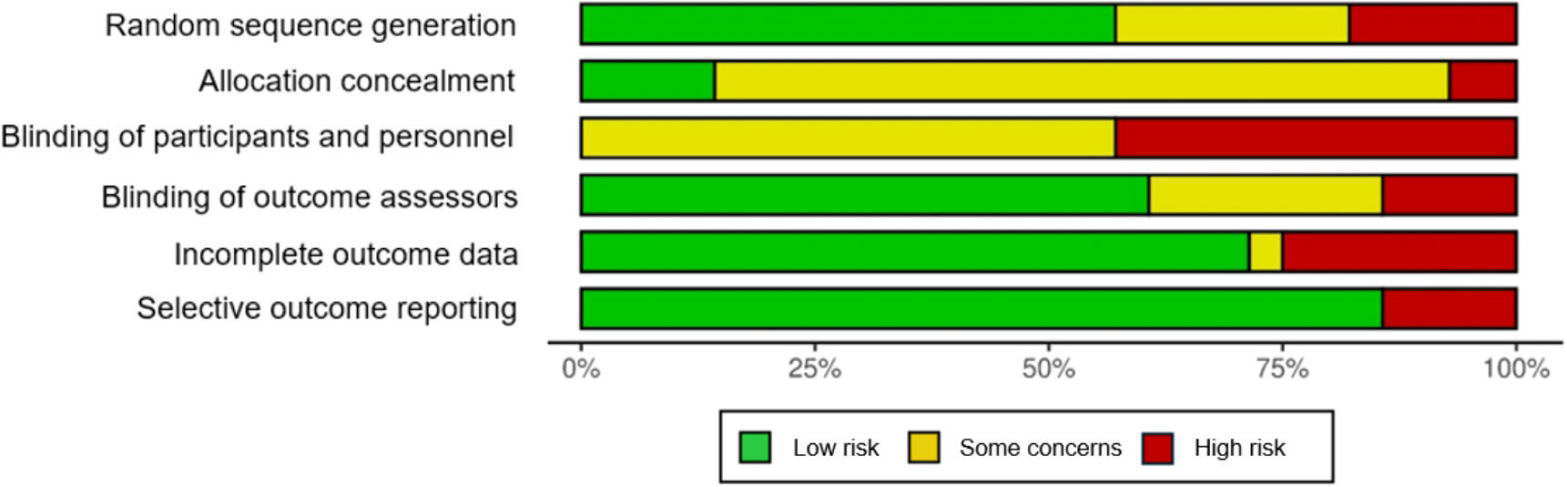
Risk of bias summary of included studies (46).

The studies exhibiting the highest number of domains rated as high risk of bias were those referenced in (51, 54, 55, 58, 60, 80). All six studies presented a high risk of bias for blinding of participants and personnel, while four demonstrated unclear or high risk of bias for allocation concealment. Additionally, three studies showed a high risk of bias for incomplete outcome data, and the study in (51) exhibited a high risk of bias in random sequence generation (See **Figure 4**).

## Discussion

### Summary of findings

This systematic review evaluated the effectiveness of resilience-based interventions designed to improve mental health outcomes in secondary school children aged 11 to 19 years. Twenty-seven studies had short-term follow-up (<6 months), while seven included long-term follow-up, ranging from 26 weeks to 3 years. Across the studies included in the review, predominantly non-significant effects were demonstrated. However, several interventions showed statistically significant effects varying by mental health outcome and length of follow-up. When analysing the results across the thirty-four studies, twelve interventions compared to a control or alternative intervention were effective for 5 of 9 outcomes, namely, depressive symptoms (49, 51, 70, 75), anxiety symptoms (63, 73, 81), resilience (53, 56, 57, 75, 82), emotional symptoms (76), and externalising problems (75).

While ten interventions demonstrated promising short-term effects, often with small effect sizes, this review revealed a lack of consistent, long-term effects across most interventions, suggesting that they were not effective in producing lasting improvements in mental health outcomes. Although interventions demonstrated short-term effectiveness for depressive symptoms, anxiety symptoms, and resilience, the long-term follow-up revealed sustained benefits only for depressive and emotional symptoms. Interventions with greater long-term effects often used a group-based format and demonstrated higher participant engagement and stronger implementation fidelity. In contrast, less effective interventions commonly faced challenges such as poor engagement and high dropout rates, often due to student transfers, absences on assessment days, or competing school activities that hindered participation and follow-up (54, 55, 58, 63, 77, 79).

Results of the studies showed that most of the interventions aimed to improve mental health outcomes used a combined approach with more than one technique or component on their program. The findings of the present review are consistent with meta-analyses by Dray et al. (18) and Pinto et al. (27), as the current synthesis confirms small-to-moderate short-term improvements in depressive symptoms, anxiety, and resilience following school-based interventions. However, we identified substantial variability in effectiveness across outcomes, populations, and follow-up durations, with limited evidence of sustained long-term effects. While Llistosella et al. (26) reported larger short-term effects for multicomponent interventions, particularly among at-risk adolescents, the present review focuses on universal, school-based interventions and suggests that observed benefits are strongly influenced by implementation quality and participant engagement. Nevertheless, in line with Llistosella’s findings, most effective interventions in the present review were multicomponent in nature, with 4 of the 8 effective interventions combining approaches such as positive psychology, cognitive behavioural therapy, and social learning theory.

Another key finding relates to the most reported component in effective interventions: social skills training. Such interventions were implemented across diverse populations, including secondary school students in Germany (76), the United States (56), Hong Kong (57), and socioeconomically disadvantaged female students in Bihar, India (63). From a theoretical perspective, social skills training may enhance resilience by strengthening interacting socio-emotional and interpersonal processes, such as social competence and utilisation of social support, which Fritz et al. conceptualise as dynamic protective mechanisms that operate in combination to support adaptive functioning under stress (83). Two studies included in this review provide evidence that social skills training can enhance resilience by fostering socio-emotional competencies such as self-awareness, social awareness, relationship skills, and responsible decision-making (84). In a randomised controlled trial, Ho et al. evaluated a group-based sports program designed to promote life skills and social connectedness among adolescents aged 12–19. The intervention produced a small but statistically significant improvement in resilience (57). However, the follow-up period was limited to four weeks, limiting inferences about long-term effects. Similarly, Green et al. conducted an RCT in a US school setting that targeted personal and social skills development, reporting a large effect size in favour of resilience (56). These findings suggest that interventions promoting socio-emotional development may strengthen peer relationships and support networks, which are recognised as key protective factors in fostering resilience (85).

Teacher-led delivery has been identified as one of the key facilitators of the successful implementation of resilience-based interventions in secondary schools (84). This is enabled by the integration of these programs into the school curriculum and the use of a standardised format, allowing teachers to deliver the intervention consistently and in a structured manner (86). The teacher-led facilitator aligns with Askell-Williams and Murray-Harvey’s (87) suggestion of a team facilitation approach, where trained facilitators collaborate with classroom teachers to enhance the effectiveness of interventions. In addition to improving delivery, the standardised format may also enhance scalability by enabling the intervention to be replicated more easily in other schools without requiring extensive adaptation. However, factors such as the compatibility of the intervention with different national curricula, educational policies, and school resources must be carefully considered. Schools in diverse sociocultural contexts may require additional adjustments to ensure successful implementation (88). Additionally, high teacher workload is a recognised international challenge (89) that may further limit the feasibility of adopting new interventions, despite being well-designed and scalable.

These findings largely support existing theories of resilience. The short-term improvements in resilience, depressive, and anxiety symptoms, particularly in interventions with socio-emotional learning and CBT components, align with Masten and Obradović’s view of resilience as grounded in adaptive systems, such as emotional and relational domains (31). The effectiveness of multicomponent interventions and social skills training also supports Grotberg’s perspective that resilience can be proactively developed (30). However, the limited evidence of sustained long-term effects across most interventions suggests that the mechanisms that help maintain resilience over time are poorly understood and under-explored in empirical research. This indicates the need for further investigation, rather than suggesting shortcomings in existing resilience theories.

### Strengths and limitations of the evidence

Studies included in this review consistently suffered from poor reporting of the allocation concealment process. In addition, although blinding was often not possible due to the nature of the intervention, increasing the risk of bias toward finding a significant effect, no such effects were observed. This suggests that other factors, such as the type of intervention or the population involved, may have contributed to lack of significant outcome effects. Moreover, the randomisation process was not consistently implemented in studies, as seen in the study by Pannebakker et al. Of the 38 schools approached, 11 had a strong preference to participate in the intervention while only 2 wanted to serve as controls (70). These preferences were ultimately honoured rather than adhering to full random allocation. Allowing schools to self-select into groups introduces selection bias, as those opting in may have differed in motivation or readiness to engage in mental health programs (90). In Pannebakker’s cluster RCT (70), such bias could have reduced the comparability between groups, compromising the internal validity of the study. Although a small reduction in depressive symptoms was found, the effect may reflect pre-existing differences between the schools rather than the intervention itself.

Once allocation to intervention or control condition was determined, some studies then faced issues with intervention fidelity. The studies conducted by Saelid et al. (74) and Tak et al. (78) indicated that teachers who served as facilitators of the intervention did not consistently implement it in accordance with the intended protocol. Saelid et al. reported that despite teachers receiving intensive training, they frequently reduced session duration or omitted key components (74). This may have resulted in a reduced effect for participants. Challenges such as insufficient support from school administration and time constraints resulted in the incomplete delivery of the program. Similarly, Tak et al. highlighted that their findings on intervention fidelity may have been flawed because they relied only on self-report from group trainers (78). This raise concerns that deviations from the intended intervention protocol may have occurred but were underreported due to trainers providing socially desirable responses rather than fully accurate answers (91). These deviations could have contributed to the lack of effects of the programs and made it difficult to ascertain if the observed outcomes were a true reflection of the intervention effectiveness or the result of the variations in implementation. Tak et al. found that the intervention did not prevent depressive symptoms (78), while Saelid et al. observed no significant changes for either anxiety or depression (74). The lack of effect in these studies could potentially reflect implementation issues rather than intervention failure. Acknowledging this has important implications for interpreting the broader evidence base, as it suggests that positive findings from studies with more rigorous design and higher implementation fidelity are likely to be more robust indicators of intervention efficacy (92). Therefore, additional supervision mechanisms such as professional oversight or group supervision may improve fidelity (93) to prevent non-adherence to intervention protocols. This need for enhanced oversight is consistent with findings from a previous systematic review, which highlighted third-party observations as a valuable tool for independently verifying program fidelity across settings (35). Although such measures may increase the complexity and resource demands of intervention delivery, they can enhance consistency and outcomes, especially in school-based programs where teachers who serve as facilitators balance multiple responsibilities (94).

The use of a waitlist control in some studies may have mitigated ethical concerns associated with withholding potentially beneficial interventions from control groups. Of the thirty-four studies reviewed, five employed waitlist controls. This approach ensures that all participants have the opportunity to receive the intervention, reducing ethical issues related to unequal access to support.

### Strengths and limitations of the review

The exclusion of individuals with developmental disabilities and diagnosed mental health conditions in this review potentially deprives a vulnerable group of benefiting from policy changes informed by this review, being those most in need and at higher risk of experiencing anxiety, depression, and other emotional challenges. Furthermore, excluding this population could potentially fail to represent the broad spectrum of students with different needs, thereby risking the perpetuation of health inequalities. Nevertheless, this decision is based on theoretical considerations, as evidence suggests that interventions for this group may require fundamentally different approaches, so it is advisable to treat them separately in further research.

While the review focused exclusively on randomised controlled trials to ensure high internal validity, this approach may have excluded valuable quasi-experimental or mixed-method studies that are commonly conducted in educational settings. As a result, some relevant evidence on resilience-based interventions, particularly regarding feasibility, implementation, or contextual factors, may not have been captured. Future reviews could consider incorporating a broader range of study designs to provide a more comprehensive understanding of the effectiveness and practical application of school-based resilience interventions.

Due to substantial heterogeneity across the included studies, it was not possible to conduct a meta-analysis as originally planned. In particular, a quantitative synthesis examining the effects of follow-up duration was not feasible due to variability in follow-up time points, inconsistent reporting of post-intervention and follow-up outcomes, and the use of diverse outcome measures across studies. While this heterogeneity limited quantitative synthesis, the inclusion of a wide range of mental health outcomes can be considered a strength of the present review, offering a broader perspective on the effects of resilience-based interventions. Nevertheless, the emphasis on commonly reported indicators may have resulted in an underrepresentation of positive psychological outcomes, such as life satisfaction, self-esteem, and broader measures of positive mental health, despite their conceptual relevance to resilience. Importantly, the examination of intervention effects across both short- and longer-term follow-up periods provides valuable insight into the sustainability of intervention effects over time.

Additionally, a notable strength of this review is its exploration of the facilitators and barriers to implementing resilience-based interventions. This aspect, which has not been thoroughly explored in previous reviews to our best knowledge, is critical for understanding the practical challenges and enablers in real-world settings. By identifying these key factors, the review offers valuable insights into the applicability and scalability of resilience-based interventions. These insights can serve as a guide for developing and adapting future programs, helping decision-makers to anticipate and overcome potential challenges in the implementation process.

### Implications of the evidence

This review supports the implementation of resilience-based interventions to reduce symptoms of depression, anxiety, and emotional distress while strengthening resilience and preventing future mental health issues among secondary school children. Given the increasing prevalence of mental health problems within this age group, it is crucial to adopt comprehensive strategies that not only address immediate emotional difficulties but also build long-term resilience as a means of promoting and sustaining overall mental well-being.

One promising approach highlighted in this review is the integration of social skills training within resilience intervention programs. Developing and enhancing social skills can significantly improve students’ ability to navigate interpersonal relationships, effectively manage conflicts, and seek support from peers and adults, thereby increasing their overall resilience to stressors (95). Moreover, focusing on these skills can reduce feelings of isolation and foster a sense of community and belonging, further mitigating feelings of anxiety and depression.

The findings of this review provide valuable insights that can guide secondary school administrators, educators, and mental health professionals in designing and enhancing resilience training programs. It is crucial to create supportive environments that not only foster mental well-being but also empower students to face future challenges with confidence and adaptability. The review identified social skills training as a key component in successful interventions, suggesting that programs integrating opportunities for social interaction can significantly enhance students’ resilience and ability to navigate future challenges. Integrating resilience-based programs into the formal secondary school curriculum is highly recommended to ensure all students have access to essential skills and resources that boost their resilience.

Moreover, identifying critical periods for implementing resilience-based interventions, such as the transition to university, could potentially enhance their effectiveness. Targeting these key times can maximise the impact of the intervention, providing timely support when students are most vulnerable to stress. This strategic approach can also increase the return on investment by optimising resource allocation and intervention outcomes.

Furthermore, these efforts could have a positive influence on prevention strategies and policymaking. Secondary schools can advocate for broader policy changes that prioritise mental health education, which can lead to improvements in student support services and contribute to the development of comprehensive mental health strategies within the educational framework, paving the way for healthier and more resilient future generations.

### Future research

Future research should prioritise addressing the methodological limitations identified in this review. Ensuring rigorous randomisation procedures and transparent reporting is critical, as deviations can compromise internal validity. Greater attention should also be given to the accurate assessment of intervention fidelity. Current reliance on self-reported adherence may introduce bias. Therefore, future evaluations should incorporate independent monitoring strategies, such as third-party observations or structured fidelity checklists. In cases where blinding of participants or facilitators is not feasible due to the nature of the intervention, it remains essential to blind outcome assessors to minimise the risk of bias. Addressing these methodological weaknesses will enhance the reliability of findings and support more robust conclusions about the effectiveness of resilience-based interventions. Building on this, future studies should also expand their target populations to include individuals with developmental disabilities and diagnosed mental health conditions. Their exclusion limits understanding of how these interventions affect vulnerable groups who may stand to benefit the most. Including such populations is crucial not only to promote equitable access but also to assess intervention effectiveness across a broader spectrum of needs.

Furthermore, cultural considerations should be more integrated into the development and evaluation of resilience-based programs. For example, mindfulness components, commonly included in these interventions, may not be equally acceptable across cultural contexts. In a pilot study conducted in Hong Kong, Lam (96) found that students were reluctant to close their eyes during meditation, citing discomfort and unfamiliarity with the practice. This highlights the importance of culturally adaptive strategies that foster student engagement and optimise outcomes in diverse school settings.

To strengthen future systematic reviews, greater consistency in the reporting of intervention components and implementation characteristics is needed. Reviews would benefit from clearly identifying and comparing key intervention components, delivery approaches, and levels of implementation fidelity, thereby advancing understanding of how resilience-based interventions are effective in real-world school settings.

Finally, the persistent lack of long-term follow-up remains a critical gap that needs to be addressed. While it is acknowledged that sustaining implementation and collecting follow-up data pose significant challenges, understanding the durability of intervention effects over time is essential. Future systematic reviews would benefit from focusing on adequately powered trials that employ standardised follow-up time points and outcome measures, to facilitate the assessment of the relationship between follow-up duration and intervention effectiveness through meta-analysis. Long-term evaluation is key to determining the sustained impact of resilience-based approaches and informing their scalability and integration into routine practice.

Beyond methodological considerations, future research should consider what resilience-based interventions mean in practice for schools and those delivering them. This should include examining whether interventions can realistically be incorporated into everyday school routines, align with existing curricula and be delivered with the necessary training and support for staff. For policymakers, greater attention to scalability, sustainability, and equity is essential to ensure that effective interventions can be adopted and maintained in diverse school settings.

## Conclusion

This review has provided a comprehensive evaluation of resilience-based interventions aimed at improving mental health outcomes and increasing resilience among secondary school children aged 11 to 19 years. While many studies reported non-significant effects, some interventions demonstrated statistically significant effects in outcomes such as depressive symptoms, anxiety symptoms, resilience, emotional symptoms, and externalising problems. A key insight from the review is that multi-component approaches, particularly those integrating social skills training along with cognitive behavioural therapy and positive psychology, are often effective.

Future research should explore how to make resilience-based interventions more culturally sensitive and inclusive of diverse populations, including those with developmental disabilities and diagnosed mental health conditions. It should also ensure long-term follow-up to assess sustainability.
