# Supplementary material for: Effectiveness of resilience-based interventions to promote mental well-being among secondary school children: a systematic review
Source: Front Psychiatry. 2026 Mar 5;17:1642660. doi: 10.3389/fpsyt.2026.1642660 (PMC13000754; doi:10.3389/fpsyt.2026.1642660)
Supplement: Supplementary file 2 [file SupplementaryFile2.docx]

**Additional file 2.** Search terms

| Ovid MEDLINE(R) 2014 to November Week 4 2025 |
| --- |
| 1. mental health.mp. or Mental Health/ 2. Stress, Psychological/ or psychological distress.mp. 3. mental hygiene.mp. 4. psychological status.mp. 5. (psychological adj (wellbeing or well being or health)).mp. 6. psychopathology.mp. or Psychopathology/ 7. (psychological* adj (stress* or adapt*)).mp. 8. mental* ill*.mp. or Mentally Ill Persons/ 9. (emotional* adj (distress* or health or wellbeing or well being)).mp. 10. exp Mental Disorders/ or mental disorder*.mp. 11. 1 or 2 or 3 or 4 or 5 or 6 or 7 or 8 or 9 or 10 12. School Health Services/ or school*.mp. 13. school based.mp. 14. classroom.mp. 15. (school* adj3 (intervention* or program* or course* or polic* or practice* or curricul* or environment*)).mp. 16. schoolchild*.mp. 17. 12 or 13 or 14 or 15 or 16 18. resilien*.mp. or Psychological Resilience/ 19. (factor* adj (protect* or promoti* or external or internal or environment*)).mp. 20. (strength* adj (based or focused)).mp. 21. (development adj3 (adolescen* or youth or child* or positive)).mp. 22. (coping or adaptability or life skill* or lifeskill* or social skill*).mp. 23. (competence adj3 (emotional or behavio?ral or social or cognitive)).mp. 24. psychological adaptation.mp. or Adaptation, Psychological/ 25. (positive psychology or psychosocial or psycho social or positive education).mp. 26. 18 or 19 or 20 or 21 or 22 or 23 or 24 or 25 27. randomized controlled trial.pt. 28. controlled clinical trial.pt. 29. random*.tw. 30. placebo.tw. 31. clinical trials as topic.sh. 32. trial.tw. 33. 27 or 28 or 29 or 30 or 31 or 32 34. 11 and 17 and 26 and 33 35. limit 34 to yr = "2014 -Current" 36. (animals not (humans and animals)).sh. 37. 35 not 36 |

| Ovid EMBASE 2014 to November Week 4 2025 |
| --- |
| 1. mental health/ or mental health.ti,ab. 2. mental stress/ or mental stress.ti,ab. 3. mental hygiene.ti,ab. 4. psychological status.ti,ab. 5. (psychological adj (wellbeing or well being or health)).ti,ab. 6. Psychopathology/ or psychopathology.ti,ab.  7. (psychological* adj (stress* or adapt*)).ti,ab. 8. mental patient/ or mental patient.ti,ab. 9. (emotional* adj (distress* or health or wellbeing or well being)).ti,ab. 10. Exp mental disease/ or mental disease.ti,ab. 11. 1 or 2 or 3 or 4 or 5 or 6 or 7 or 8 or 9 or 10 12. school health service/ or school*.ti,ab. 13. school based.ti,ab. 14. classroom.ti,ab. 15. (school* adj3 (intervention* or program* or course* or polic* or practice* or curricul* or environment*)).ti,ab. 16. schoolchild*.ti,ab. 17. 12 or 13 or 14 or 15 or 16 18. psychological resilience/ or resilien*.ti,ab. 19. (factor* adj (protect* or promoti* or external or internal or environment*)).ti,ab. 20. (strength* adj (based or focused)).ti,ab. 21. (development adj3 (adolescen* or youth or child* or positive)).ti,ab. 22. (coping or adaptability or life skill* or lifeskill* or social skill*).ti,ab. 23. (competence adj3 (emotional or behavio?ral or social or cognitive)).ti,ab. 24. adaptation, psychological/ or psychological adjustment.ti,ab. 25. (positive psychology or psychosocial or psycho social or positive education).ti,ab. 26. 18 or 19 or 20 or 21 or 22 or 23 or 24 or 25 27. randomized controlled trial/ 28. controlled clinical trial/ 29. random$.ti,ab. 30. placebo.ti,ab. 31. trial.tw. 32. 27 or 28 or 29 or 30 or 31 33. 11 and 17 and 26 and 32 34. limit 33 to yr="2014- Current" 35. animals/ not (humans/ and animals/) 36. 34 not 35 |

| Ovid PsycINFO 2014 to November Week 4 2025 |
| --- |
| 1. Mental Health/ or mental health.ti,ab.  2. Psychological Stress/ or psychological distress.ti,ab.  3. mental hygiene.ti,ab.  4. psychological status.ti,ab.  5. (psychological adj (wellbeing or well being or health)).ti,ab.  6. Psychopathology/ or psychopathology.ti,ab.  7. (psychological* adj (stress* or adapt*)).ti,ab.  8. mental* ill*.ti,ab. or mentally ill persons/  9. (emotional* adj (distress* or health or wellbeing or well being)).ti,ab.  10. exp Mental Disorders/ or mental disorder*.ti,ab.  11. 1 or 2 or 3 or 4 or 5 or 6 or 7 or 8 or 9 or 10  12. school health services/ or school*.ti,ab.  13. school based.ti,ab.  14. classroom.ti,ab.  15. (school* adj3 (intervention* or program* or course* or polic* or practice* or curricul* or environment*)).ti,ab.  16. schoolchild*.ti,ab.  17. 12 or 13 or 14 or 15 or 16  18. Coping Behavior/ or resilien*.ti,ab.  19. (factor* adj (protect* or promoti* or external or internal or environment*)).ti,ab.  20. (strength* adj (based or focused)).ti,ab.  21. (development adj3 (adolescen* or youth or child* or positive)).ti,ab.  22. (coping or adaptability or life skill* or lifeskill* or social skill*).ti,ab.  23. (competence adj3 (emotional or behavio?ral or social or cognitive)).ti,ab.  24. Emotional Adjustment/ or psychological adaptation.ti,ab.  25. (positive psychology or psychosocial or psycho social or positive education).ti,ab.  26. 18 or 19 or 20 or 21 or 22 or 23 or 24 or 25  27. Randomized Controlled Trials/  28. Clinical Trials/  29. random$.ti,ab.  30. placebo.ti,ab.  31. trial.tw.  32. 27 or 28 or 29 or 30 or 31  33. 11 and 17 and 26 and 32  34. limit 33 to yr="2014 - Current"  35. animals/ not (humans/ and animals/) 36. 34 not 35 |

| CINAHL 2014 to November Week 4 2025 |
| --- |
| S1. MH "Mental Health"  S2. TI “mental health” or AB “mental health”  S3. MH "Psychological Stress" S4. TI "psychological stress" or AB “psychological stress”  S5. TI "psychological distress" or AB “psychological distress”  S6. TI “mental hygiene” or AB “mental hygiene”  S7. TI "psychological status" or AB "psychological status"  S8. TI ("psychological" N3 ("wellbeing" or "well being" or "health")) or AB ("psychological" N3 ("wellbeing" or "well being" or "health"))  S9. MH "Psychopathology"  S10. TI "psychopathology" or AB "psychopathology"  S11. TI ("psychological*" N3 ("stress*" or "adapt*")) or AB ("psychological*" N3 ("stress*" or "adapt*")) S12. MH "Mentally Ill Persons"  S13. TI "mentally ill persons" or AB "mentally ill persons"  S14. TI ("emotional*" N3 ("distress*" or "health" or "wellbeing" or "well being")) or AB ("emotional*" N3 ("distress*" or "health" or "wellbeing" or "well being")) S15. MH "Mental Disorders+"  S16. TI "mental disorder*" or AB "mental disorder*"  S17. S1 OR S2 OR S3 OR S4 OR S5 OR S6 OR S7 OR S8 OR S9 OR S10 OR S11 OR S12 OR S13 OR S14 OR S15 OR S16  S18. MH "School Health Services"  S19. TI “school*” or AB “school*” S20. TI "school-based" or AB "school-based"  S21. TI "classroom" or AB "classroom"  S22. TI (school* N3 ("intervention*" or "program*" or "course*" or "polic*" or "practice*" or "curricul*" or "environment*")) or AB (school* N3 ("intervention*" or "program*" or "course*" or "polic*" or "practice*" or "curricul*" or "environment*"))  S23. TI "schoolchild*" or AB "schoolchild*" S24. S18 OR S19 OR S20 OR S21 OR S22 OR S23  S25. MH "Psychological Resilience"  S26. TI "resilience" or AB "resilience"  S27. TI ("factor*" N3 ("protect*" or "promot*" or "external" or "internal" or "environment*")) or AB ("factor*" N3 ("protect*" or "promot*" or "external" or "internal" or "environment*")) S28. TI ("strength*" N3 ("based" or "focused")) or AB ("strength*" N3 ("based" or "focused")) S29. TI ("development" N3 ("adolescen*" or "youth" or "child*" or "positive")) or AB ("development" N3 ("adolescen*" or "youth" or "child*" or "positive"))  S30. TI ("coping" or "adaptability" or "life skill*" or "lifeskill*" or "social skill*") or AB ("coping" or "adaptability" or "life skill*" or "lifeskill*" or "social skill*") S31. TI ("competence" N3 ("emotional" or "behavio?ral" or "social" or "cognitive")) or AB ("competence" N3 ("emotional" or "behavio?ral" or "social" or "cognitive")) S32. MH "Psychological Adjustment"  S33. TI "psychological adaptation" or AB "psychological adaptation" S34. TI ("positive psychology" or "psychosocial" or "psycho social" or "positive education") or AB ("positive psychology" or "psychosocial" or "psycho social" or "positive education").  S35. S25 OR S26 OR S27 OR S28 OR S29 OR S30 OR S31 OR S32 OR S33 OR S34  S36. MH "Randomized Controlled Trials"  S37. MH "Clinical Trials" S38. TI "random*" or AB "random*"  S39. TI "placebo" or AB "placebo" S40. TI “trial” or AB “trial”  S41. S36 OR S37 OR S38 OR S39 OR S40  S42. S17 AND S24 AND S35 AND S41  S43. Limit S42 to yr = 2014- Current S44. MH "Animals" NOT ("humans" AND "animals") S45. S43 NOT S44 |

| CENTRAL 2014 to November Week 4 2025 |
| --- |
| 1. “Mental health”:ti,ab,kw 2. “Psychological distress”:ti,ab,kw 3. “Mental hygiene”:ti,ab,kw 4. “Psychological status”:ti,ab,kw 5. (psychological NEXT (wellbeing OR "well being" OR health)):ti,ab,kw 6. “Psychopathology”:ti,ab,kw 7. (psychological* NEXT (stress* OR adapt*)):ti,ab,kw 8. “Mental* ill*”:ti,ab,kw 9. (emotional* NEXT (distress* OR health OR wellbeing OR "well being")):ti,ab,kw 10. “Mental disorder*”:ti,ab,kw 11. #1 OR #2 OR #3 OR #4 OR #5 OR #6 OR #7 OR #8 OR #9 OR #10 12. “School*”:ti,ab,kw 13. “School based”:ti,ab,kw 14. “Classroom”:ti,ab,kw 15. (school* NEXT3 (intervention* OR program* OR course* OR polic* OR practice* OR curricul* OR environment*):ti,ab,kw 16. “Schoolchild*”:ti,ab,kw 17. #12 OR #13 OR #14 OR #15 OR #16 18. “Resilien*”:ti,ab,kw 19. (factor* NEXT (protect* OR promoti* OR external OR internal OR environment*)):ti,ab,kw 20. (strength* NEXT (based OR focused)):ti,ab,kw 21. (development NEXT3 (adolescen* OR youth OR child* OR positive)):ti,ab,kw 22. (coping OR adaptability OR "life skill*" OR lifeskill* OR "social skill*"):ti,ab,kw 23. (competence NEXT3 (emotional OR behavio?ral OR social OR cognitive)):ti,ab,kw 24. “Psychological adaptation”:ti,ab,kw 25. (“positive psychology” OR psychosocial OR "psycho social" OR “positive education”):ti,ab,kw 26. #18 OR #19 OR #20 OR #21 OR #22 OR #23 OR #24 OR #25 27. #11 AND #17 AND #26 |
