# Supplementary material for: Effectiveness of resilience-based interventions to promote mental well-being among secondary school children: a systematic review
Source: Front Psychiatry. 2026 Mar 5;17:1642660. doi: 10.3389/fpsyt.2026.1642660 (PMC13000754; doi:10.3389/fpsyt.2026.1642660)
Supplement: Supplementary file 3 [file SupplementaryFile3.docx]

**Additional file 3**. List of excluded publications and reasons for exclusion

| **Publication** | **Reason for exclusion** |
| --- | --- |
| Abarca B, Dosanjh K, Dudovitz R. 4.15 MENTAL HEALTH OUTCOMES AMONG HIGH SCHOOL STUDENTS RANDOMIZED TO AVID PIPELINE PROGRAM. Journal of the American Academy of Child & Adolescent Psychiatry [Internet]. 2019 Oct 1;58(10):S224–4. | Ineligible population |
| Aber JL, Tubbs C, Torrente C, Halpin PF, Johnston B, Starkey L, et al. Promoting children’s learning and development in conflict-affected countries: Testing change process in the Democratic Republic of the Congo. Development and Psychopathology. 2016 Nov 21;29(1):53–67. | Incorrect intervention |
| Harris N. Pau te Hau - Training Teachers to Deliver High Intensity Interval Training (HIIT) to School Students [Internet]. 2021. Available from: https://www.anzctr.org.au/Trial/Registration/TrialReview.aspx?id=381219 | Incorrect study design |
| Harris N. Pau te Hau: curriculum-based high-intensity interval training for young adolescents. http://www.who.int/trialsearch/Trial2.aspx?TrialID=ACTRN12617000910303 2017;(): 2017. | Incorrect intervention |
| Kimonis. Improving mental health outcomes through parent engagement in school-based early intervention. https://trialsearch.who.int/Trial2.aspx?TrialID=ACTRN12619000967189 2019;(): 2019. | Incorrect study design |
| O’Dea et al. Examining the delivery of Smooth Sailing in Secondary Schools. https://trialsearch.who.int/Trial2.aspx?TrialID=ACTRN12621000225819 2021;(): 2021. | Incorrect intervention |
| Barrett. A school-based randomised controlled trial of a universal mental health prevention program: ourFutures Mental Health. https://trialsearch.who.int/Trial2.aspx?TrialID=ACTRN12622001582741 2022;(): 2022. | Ongoing RCT |
| Ager A, Metzler J. Where there is no intervention: Insights into processes of resilience supporting war-affected children. Peace and Conflict: Journal of Peace Psychology. 2017 Feb;23(1):67–75. | Incorrect study design |
| Akeman E, Kirlic N, Clausen AN, Cosgrove KT, McDermott TJ, Cromer LD, et al. A pragmatic clinical trial examining the impact of a resilience program on college student mental health. Depression and Anxiety. 2019 Nov 4;37(3):202–13. | Ineligible population |
| Amudhan S, Jangam K, Mani K, Murugappan NP, Sharma E, Mahapatra P, et al. Project SUMS (scaling up of mental health in schools): design and methods for a pragmatic, cluster randomised waitlist-controlled trial on integrated school mental health intervention for adolescents. BMC Public Health [Internet]. 2021 Nov 6;21(1). | Incorrect study design |
| Bazzano AN, Sun Y, Chavez-Gray V, Akintimehin T, Gustat J, Barrera D, et al. Effect of Yoga and Mindfulness Intervention on Symptoms of Anxiety and Depression in Young Adolescents Attending Middle School: A Pragmatic Community-Based Cluster Randomized Controlled Trial in a Racially Diverse Urban Setting. International Journal of Environmental Research and Public Health [Internet]. 2022 Jan 1;19(19):12076. | Ineligible population |
| Birrell L, Debenham J, Ainsley Furneaux-Bate, Prior K, Spallek S, Thornton L, et al. Evaluating a Peer-Support Mobile App for Mental Health and Substance Use Among Adolescents Over 12 Months During the COVID-19 Pandemic: Randomized Controlled Trial. JMIR Journal of medical internet research/Journal of medical internet research [Internet]. 2023 Sep 27;25:e45216–6. | Incorrect intervention |
| Bleasdale JE, Peterson MC, Nidich S. Effect of Meditation on Social/Emotional Well-Being in a High-Performing High School. Professional School Counseling. 2019 Jan;23(1):2156759X2094063. | Incorrect study design |
| Bluth K, Campo RA, Pruteanu-Malinici S, Reams A, Mullarkey M, Broderick PC. A school-based mindfulness pilot study for ethnically diverse at-risk adolescents. Mindfulness. 2016 Jan 4;7(1):90–104. | Incorrect study design |
| Bonell C, Allen E, Warren E, McGowan J, Bevilacqua L, LeGood R, et al. A Multi-Component School Environment Intervention Reduces Bullying and Risky Behaviour and Improves Mental Health and Quality of Life: Findings From the INCLUSIVE Cluster Randomized Controlled Trial. Journal of Adolescent Health. 2018 Feb;62(2):S9. | Incorrect study design |
| Boucher E, Ward H, Miles C, Henry RD, Sarah Elizabeth Stoeckl. Effects of a Digital Mental Health Intervention on Perceived Stress and Rumination in Adolescents Aged 13 to 17: A Randomized Controlled Trial (Preprint). JMIR Journal of medical internet research/Journal of medical internet research. 2024 Mar 29;26:e54282–2. | Ineligible population |
| Britton et al. A randomized controlled pilot trial of classroom-based mindfulness meditation compared to an active control condition in sixth-grade children. https://www.sciencedirect.com/science/article/abs/pii/S0022440514000296?via%3Dihub. 2014. | Incorrect study design |
| Brown CL, Christian DD, H. Michael Crowson. Effects of an Adventure Therapy Mountain Bike Program on Middle School Students’ Resiliency. Journal of Child and Adolescent Counseling. 2023 May 4;9(2):222–41. | Incorrect intervention |
| Brunwasser SM, Freres DR, Gillham JE. Youth Cognitive-Behavioral Depression Prevention: Testing Theory in a Randomized Controlled Trial. Cognitive Therapy and Research. 2018 Feb 21;42(4):468–82. | Incorrect outcomes |
| Byansi et al. The Short-Term Impact of a Combination Intervention on Depressive Symptoms Among School-Going Adolescent Girls in Southwestern Uganda: The Suubi4Her Cluster Randomized Trial. https://linkinghub.elsevier.com/retrieve/pii/S1054139X22004165. 2022. | Incorrect study design |
| Carreres-Ponsoda, F., Escartí, A., Llopis-Goig, R., & Cortell-Tormo, J. M. (2017). The effect of an out-of-school mindfulness program on adolescents’ stress reduction and emotional wellbeing. *Cuadernos de Psicología del Deporte, 17*(3), 35–44. | Incorrect outcomes |
| Chandrasekhar JL, Bowen AE, Heberlein E, Pyle E, Studts CR, Simon SL, et al. Universal, School-Based Mental Health Program Implemented Among Racially and Ethnically Diverse Youth Yields Equitable Outcomes: Building Resilience for Healthy Kids. Community Mental Health Journal. 2023 Feb 9; | Incorrect study design |
| Chen et al. The influence of middle school students’ physical exercise on their coping style: The mediating role of mental resilience. https://www.cochranelibrary.com/central/doi/10.1002/central/CN-02095017/full. 2019. | Incorrect intervention |
| Cheng, N. EFFECT OF MARTIAL ARTS TRAINING ON THE PHYSICAL AND MENTAL HEALTH OF YOUNG PEOPLE. Psychiatria Danubina, 2021; Vol. 33, Suppl. 5, pp 7-240. 2021. | Incorrect outcomes |
| Chung JOK, Li WHC, Ho KY, Lam KKW, Cheung AT, Ho LLK, et al. Adventure‐based training to enhance resilience and reduce depressive symptoms among juveniles: A randomized controlled trial. Research in Nursing & Health. 2021 Mar 23;44(3):438–48. | Incorrect setting |
| Cook CR, Frye M, Slemrod T, Lyon AR, Renshaw TL, Zhang Y. An integrated approach to universal prevention: Independent and combined effects of PBIS and SEL on youths’ mental health. School Psychology Quarterly. 2015 Jun;30(2):166–83. | Incorrect study design |
| Dray J, Bowman J, Freund M, Campbell E, Wolfenden L, Hodder RK, et al. Improving adolescent mental health and resilience through a resilience-based intervention in schools: study protocol for a randomised controlled trial. Trials. 2014 Jul 18;15(1). | Incorrect study design |
| Duagi, D. Evaluating the long-term outcomes of a school-based intervention for depression in older adolescents. Brain and Neuroscience Advances 2023;7():64. 2023. | Incorrect study design |
| Eather N, Morgan PJ, Lubans DR. Effects of exercise on mental health outcomes in adolescents: Findings from the crossfit^TM^ teens randomized controlled trial. Psychology of Sport and Exercise. 2016 Sep;26(1):14–23. | Incorrect intervention |
| Eschenbeck H, Lehner L, Hofmann H, Bauer S, Becker K, Diestelkamp S, et al. School-based mental health promotion in children and adolescents with StresSOS using online or face-to-face interventions: study protocol for a randomized controlled trial within the ProHEAD Consortium. Trials. 2019 Jan 18;20(1). | Incorrect study design |
| Fahey et al. Rationale and design of Peer Engagement to Enhance Resiliency (PEER) program to improve preconception mental health among Indian adolescents. Archives of Women’s Mental Health 2019;22(5):679. | Incorrect study design |
| Filiatreau LM, Nhial Tutlam, Brathwaite R, Byansi W, Namuwonge F, Mwebembezi A, et al. Effects of a Combination Economic Empowerment and Family Strengthening Intervention on Psychosocial Well-being Among Ugandan Adolescent Girls and Young Women: Analysis of a Cluster Randomized Controlled Trial (Suubi4Her). 2023 May 1;72(5):S33–40. | Incorrect intervention |
| Ford et al. The Role of Schools in Early Adolescents’ Mental Health: Findings From the MYRIAD Study. Journal of the American Academy of Child and Adolescent Psychiatry 2021;60(12):1467-1478. 2021. | Incorrect study design |
| Frank JL, Kohler K, Peal A, Bose B. Effectiveness of a School-Based Yoga Program on Adolescent Mental Health and School Performance: Findings from a Randomized Controlled Trial. Mindfulness. 2016 Oct 25;8(3):544–53. | Incorrect study design |
| Frazier SL, Dinizulu SM, Rusch D, Boustani MM, Mehta TG, Reitz K. Building Resilience After School for Early Adolescents in Urban Poverty: Open Trial of Leaders @ Play. Administration and Policy in Mental Health and Mental Health Services Research [Internet]. 2014 Nov 26;42(6):723–36. | Incorrect setting |
| Gigantesco A, Del Re D, Cascavilla I, Palumbo G, De Mei B, Cattaneo C, et al. A Universal Mental Health Promotion Programme for Young People in Italy. BioMed Research International. 2015;2015:1–9. | Incorrect study design |
| Gijzen M, Rasing S, van den Boogaart R, Rongen W, van der Steen T, Creemers D, et al. Feasibility of a serious game coupled with a contact-based session led by lived experience workers for depression prevention in high-school students. Mordaunt DA, editor. PLOS ONE. 2021 Nov 30;16(11):e0260224. | Incorrect outcomes |
| Goyal P, Chandra M, None Rushi, Choudhary M. The Effect of Mindfulness Practice on Adolescents: A Pilot Study. Journal of Indian Association for Child and Adolescent Mental Health. 2023 Sep 18;19(2). | Incorrect study design |
| Green AL, Ferrante S, Boaz TL, Kutash K, Wheeldon‐Reece B. Social and emotional learning during early adolescence: Effectiveness of a classroom‐based SEL program for middle school students. Psychology in the Schools. 2021 Feb;58(6). | Incorrect study design |
| Hamdani SU, Warraitch A, Suleman N, Muzzafar N, Minhas FA, Nizami AT, et al. Technology-Assisted Teachers’ Training to Promote Socioemotional Well-Being of Children in Public Schools in Rural Pakistan. Psychiatric Services. 2021 Jan 1;72(1):69–76. | Ineligible population |
| Tahere Harooni, Zarabian MK, Mousavi AS. The Effect of an Anger Control Training Program on Resilience, Hardiness and General Health of School Girls. Iranian Journal of Psychiatry and Clinical Psychology [Internet]. 2020 Apr 30;368–83. | Incorrect study design |
| Henin et al. 31.2 Teaching Resiliency Skills to Children and Adolescents: A Pilot Study. 2021, editor. Journal of the American Academy of Child & Adolescent Psychiatry, Volume 60, Issue 10, S212. | Incorrect study design |
| Hides L, Pocuca N, Quinn C, Kavanagh D, Johnson D. The RAW wellbeing program for adolescents at risk of disengaging from education, employment, and training. ˜The œjournal of positive psychology. 2024 Feb 27;1–11. | Incorrect study design |
| Hodas R. An investigation of the relationship between positive and negative mental health factors and academic performance among early adolescent girls. Dissertation Abstracts International: Section B: The Sciences and Engineering 2016;76(12-B(E)). 2016. | Incorrect study design |
| Tomlinson M. Project HASHTAG: testing a school-based intervention to improve adolescent mental health in Nepal and South Africa. https://trialsearch.who.int/Trial2.aspx?TrialID=ISRCTN80690743 2021;(): 2021. | Incorrect study design |
| Carli et al. Evaluating the Youth Aware of Mental Health (YAM): a mental health promotion program for schools. https://trialsearch.who.int/Trial2.aspx?TrialID=ISRCTN17583138 2018;(): 2018. | Incorrect intervention |
| Daniel M. Evaluating the effectiveness of the psychosocial, movement-based intervention TeamUp in Burundi. https://trialsearch.who.int/Trial2.aspx?TrialID=ISRCTN17499603 2023;(): 2023. | Incorrect intervention |
| Janssen TWP, van Atteveldt N. Coping styles mediate the relation between mindset and academic resilience in adolescents during the COVID-19 pandemic: a randomized controlled trial. Scientific Reports. 2023 Apr 13;13(1). | Incorrect outcomes |
| Juul L, Frydenberg M, Beck MS, Fjorback LO. Stress-free Everyday LiFe for Children and Adolescents REsearch (SELFCARE): a protocol for a cluster randomised trial testing a school teacher training programme to teach mindfulness (“.b”). BMC Psychology. 2021 Feb 17;9(1). | Incorrect study design |
| Katz J, Mercer SH, Skinner S. Developing Self-concept, Coping Skills, and Social Support in Grades 3–12: A Cluster-Randomized Trial of a Combined Mental Health Literacy and Dialectical Behavior Therapy Skills Program. School Mental Health. 2019 Dec 14; | Ineligible population |
| Kelley T, Kessel A, Collings R, Rubenstein B, Monnickendam C, Solomon A. Evaluation of the iHEART mental health education programme on resilience and well-being of UK secondary school adolescents. Journal of Public Mental Health. 2021 Jan 11;20(1):43–50. | Incorrect study design |
| Khanna P, Singh K. Do All Positive Psychology Exercises Work for Everyone? Replication of Seligman et al.’s (2005) Interventions among Adolescents. Psychological Studies. 2019 Mar;64(1):1–10. | Incorrect intervention |
| Khanna P, Singh K, Proctor C. Exploring the Impact of a Character Strengths Intervention on Well-Being in Indian Classrooms. School Mental Health. 2021 Apr 27;13. | Incorrect outcomes |
| Kilbourne AM, Smith SN, Choi SY, Koschmann E, Liebrecht C, Rusch A, et al. Adaptive School-based Implementation of CBT (ASIC): clustered-SMART for building an optimized adaptive implementation intervention to improve uptake of mental health interventions in schools. Implementation Science. 2018 Sep 5;13(1). | Ineligible population |
| Klim-Conforti P, Levitt AJ, Cheung AH, Loureiro R, Fefergrad M, Schaffer A, et al. Youth voices and experiences regarding a school-based cognitive behavioral therapy skills intervention: lessons for future engagement and adaptation. BMC Public Health. 2022 Sep 9;22(1). | Incorrect outcomes |
| Kumkun et al. Effect of A Resilience Programme Through Group Dynamics on the Academic Problems of Grade 7 Students, Chiang Mai University Demonstration School. https://openpsychologyjournal.com/VOLUME/15/ELOCATOR/e187435012206100/. 2022. | Incorrect study design |
| Kvalø SE, Natlandsmyr IK. The effect of physical-activity intervention on children’s health-related quality of life. Scandinavian Journal of Public Health. 2020 Nov 24;140349482097149. | Incorrect intervention |
| Lakes et al. I am me: Adolescent perspectives of a school-based universal intervention program designed to promote emotional competence. Special Issue: Qualitative Research on Children’s Well-being Across National and Cultural Contexts. 2019;11(1):97-114. 2019. | Incorrect study design |
| Las Hayas C, Izco-Basurko I, Fullaondo A, Gabrielli S, Zwiefka A, Hjemdal O, et al. UPRIGHT, a resilience-based intervention to promote mental well-being in schools: study rationale and methodology for a European randomized controlled trial. BMC Public Health. 2019 Oct 29;19(1). | Incorrect study design |
| Lee JA, Heberlein E, Pyle E, Caughlan T, Rahaman D, Sabin M, et al. Study protocol for a school-based single group pragmatic trial to promote resilience in youth: Building Resilience for Healthy Kids. Contemporary Clinical Trials Communications. 2021 Mar;21:100721. | Incorrect study design |
| Li L. EFFECT OF BASKETBALL ON COLLEGE STUDENTS’ MENTAL AND EMOTIONAL STRESS INDUCED BY EXERCISE. Psychiatria Danubina 2021;33(Supplement 7):S437-S438 2021. 2021. | Ineligible population |
| Li W. Promoting Mental Well-being for Secondary School Students Through an Experiential Learning Activity. https://clinicaltrials.gov/show/NCT04329052 2020;(): 2020. | Incorrect intervention |
| Mascayano F, Schilling S, Tapia E, Santander F, Burrone MS, Yang LH, et al. Using Information and Communication Technologies to Prevent Suicide Among Secondary School Students in Two Regions of Chile: A Randomized Controlled Trial. Frontiers in Psychiatry. 2018 Jun 5;9. | Incorrect study design |
| McArthur et al. Change processes in school-based humanistic counselling. Counselling & Psychotherapy Research 2016;16(2):88-99. 2016. | Incorrect study design |
| McMullen J, McMullen N. Evaluation of a teacher-led, life-skills intervention for secondary school students in Uganda. Social Science and Medicine 2018;217():10-17. 2018. | Incorrect study design |
| Mertens ECA, Deković M, Van Londen M, Reitz E. Personality as a moderator of intervention effects: Examining differential susceptibility. Personality and Individual Differences. 2022 Feb;186:111323. | Incorrect intervention |
| Montero-Marin J, Allwood M, Ball S, Crane C, De Wilde K, Hinze V, et al. School-based mindfulness training in early adolescence: what works, for whom and how in the MYRIAD trial? Evidence Based Mental Health. 2022 Jul 12;25(3):117–24. | Incorrect study design |
| Montero-Marin J, Nuthall E, Byford S, Crane C, Dalgleish T, Ford T, et al. Update to the effectiveness and cost-effectiveness of a mindfulness training programme in schools compared with normal school provision (MYRIAD): study protocol for a randomised controlled trial. Trials. 2021 Apr 7;22(1). | Incorrect study design |
| Moore B, Dudley D, Woodcock S. The effects of martial arts participation on mental and psychosocial health outcomes: a randomised controlled trial of a secondary school-based mental health promotion program. BMC Psychology [Internet]. 2019 Sep 11;7(60). | Incorrect study design |
| Moore JA. Examination of the effects of computer assisted mindfulness strategies with adolescents in an alternative high school setting. Dissertation Abstracts International: Section B: The Sciences and Engineering 2018;79(5-B(E)):No-Specified. 2018. | Incorrect study design |
| Mowatt HS. School-based implementation of a prevention of depression program with urban at-risk adolescents. Dissertation Abstracts International Section A: Humanities and Social Sciences 2018;79(5-A(E)):No-Specified. 2018. | Incorrect study design |
| Muratori P, Bertacchi I, Masi G, Milone A, Nocentini A, Powell NP, et al. Effects of a universal prevention program on externalizing behaviors: Exploring the generalizability of findings across school and home settings. Journal of School Psychology [Internet]. 2019 Dec 1;77:13–23. | Ineligible population |
| Raes F. Effects of Mindfulness Training on the Emotional Experience and (Non-) Acceptance of Emotions in Adolescents. https://clinicaltrials.gov/show/NCT04159272 2019;(): 2019. | Incorrect intervention |
| Stabelini et al. A Physical Activity Program for Adolescents - ActTeens. https://clinicaltrials.gov/show/NCT05070377 2021;(): 2021. | Incorrect intervention |
| Olowokere AE, Okanlawon FA. The Effects of a School-Based Psychosocial Intervention on Resilience and Health Outcomes Among Vulnerable Children. The Journal of School Nursing. 2013 Aug 20;30(3):206–15. | Incorrect study design |
| Ordonez et al. 15.1 CONTROLLED TRIAL OF AN EVIDENCE-BASED PROGRAM TO BUILD RESILIENCE IN AT-RISK ADOLESCENTS IN COLOMBIA. Journal of the American Academy of Child and Adolescent Psychiatry 2019;58(10 Supplement):S21-S22. 2019. | Incorrect intervention |
| Peltonen K, Aalto S, Vänskä M, Lepistö R, Punamäki RL, Soye E, et al. Effectiveness of Promotive and Preventive Psychosocial Interventions on Improving the Mental Health of Finnish-Born and Immigrant Adolescents. International Journal of Environmental Research and Public Health. 2022 Mar 20;19(6):3686. | Incorrect intervention |
| Perkins AM, Bowers G, Cassidy J, Meiser‐Stedman R, Pass L. An enhanced psychological mindset intervention to promote adolescent wellbeing within educational settings: A feasibility randomized controlled trial. Journal of Clinical Psychology. 2021 Jan 15;77(4):946–67. | Incorrect study design |
| Perry Y, Petrie K, Buckley H, Cavanagh L, Clarke D, Winslade M, et al. Effects of a classroom-based educational resource on adolescent mental health literacy: A cluster randomised controlled trial. Journal of Adolescence [Internet]. 2014 Oct;37(7):1143–51. | Incorrect outcomes |
| Ponnuthurai S, Brown J. Meta-Analysis of the Outcome of RCTs of Preventative or Early Intervention Universal Group Psychological Therapies in Under 18s. Journal of the American Academy of Child and Adolescent Psychiatry 2018;57(10 Supplement):S189. 2018. | Incorrect study design |
| Rath S, Prost A, Samal S, Pradhan H, Copas A, Gagrai S, et al. Community youth teams facilitating participatory adolescent groups, youth leadership activities and livelihood promotion to improve school attendance, dietary diversity and mental health among adolescent girls in rural eastern India: protocol for a cluster-randomised controlled trial. Trials. 2020 Jan 8;21(1). | Incorrect study design |
| Ramaiya MK, McLean CL, Pokharel M, Thapa K, Schmidt MA, Berg M, et al. Feasibility and Acceptability of a School-Based Emotion Regulation Prevention Intervention (READY-Nepal) for Secondary School Students in Post-Earthquake Nepal. International Journal of Environmental Research and Public Health [Internet]. 2022 Jan 1;19(21):14497. | Incorrect study design |
| Roberts RM, Fawcett L, Searle A. An Evaluation of the Effectiveness of the Personal Leadership Program Designed to Promote Positive Outcomes for Adolescents. Journal of Happiness Studies. 2018 Mar 2;20(3):743–57. | Incorrect study design |
| Roth RA. Improving middle school students’ subjective well-being: Efficacy of a multi-component Positive Psychology Intervention targeting small groups of youth and parents. Dissertation Abstracts International: Section B: The Sciences and Engineering 2015;76(4-B(E)):No-Specified. 2015. | Incorrect study design |
| Sabin C, Bowen AE, Heberlein E, Pyle E, Lund L, Studts CR, et al. The Impact of a Universal Mental Health Intervention on Youth with Elevated Negative Affectivity: Building Resilience for Healthy Kids. Contemporary School Psychology. 2021 Jul 27; | Incorrect study design |
| Sánchez-Hernández Ó, Méndez FX, Ato M, Garber J. Prevention of Depressive Symptoms and Promotion of Well-being in Adolescents: A Randomized Controlled Trial of the Smile Program. Anales de Psicología. 2019 Apr 7;35(2):300–13. | Ineligible population |
| Shapiro AJ. Effectiveness of stress off strategies: A single-session school-based stress management program for adolescents. Dissertation Abstracts International Section A: Humanities and Social Sciences 2021;82(10-A):No-Specified. 2021. | Incorrect study design |
| Shoshani et al. Effects of the Maytiv positive psychology school program on early adolescents’ well-being, engagement, and achievement. Journal of school psychology 2016;57():73â92. 2016. | Incorrect study design |
| Simkiss NJ, Gray NS, Kemp AH, Dunne C, Robert Jefferson Snowden. A randomised controlled trial evaluating the Guide Cymru mental health literacy intervention programme in year 9 (age 13–14) school pupils in Wales. BMC Public Health. 2023 Jun 5;23(1). | Incorrect intervention |
| Singh N, Minaie MG, Skvarc DR, Toumbourou JW. Impact of a Secondary School Depression Prevention Curriculum on Adolescent Social-Emotional Skills: Evaluation of the Resilient Families Program. Journal of Youth and Adolescence. 2019 Feb 25;48(6):1100–15. | Ineligible population |
| Skoradal et al. “11 for Health” in the Faroe Islands: popularity in schoolchildren aged 10-12 and the effect on well-being and health knowledge. Scandinavian journal of medicine & science in sports 2023;33(6):1010â1020. 2023. | Incorrect intervention |
| Smedegaard S, Christiansen LB, Lund-Cramer P, Bredahl T, Skovgaard T. Improving the well-being of children and youths: a randomized multicomponent, school-based, physical activity intervention. BMC Public Health. 2016 Oct 28;16(1). | Incorrect outcomes |
| Tam CC, Li X, Benotsch EG, Lin D. A Resilience‐Based Intervention Programme to Enhance Psychological Well‐Being and Protective Factors for Rural‐to‐Urban Migrant Children in China. Applied Psychology: Health and Well-Being. 2019 Jul 9; | Ineligible population |
| Tang Y, Diao H, Jin F, Pu Y, Wang H. The effect of peer education based on adolescent health education on the resilience of children and adolescents: A cluster randomized controlled trial. Kabir E, editor. PLOS ONE. 2022 Feb 2;17(2):e0263012. | Ineligible population |
| Terry J. Preliminary evaluation of “Footprints:” Motivational interviewing to promote cognitive-behavioral skills, academic outcomes, and academic protective factors in middle school students. Dissertation Abstracts International: Section B: The Sciences and Engineering 2017;78(4-B(E)):No-Specified. 2017. | Incorrect study design |
| Theurel A, Gimbert F, Gentaz É. The effectiveness of a school-based mindfulness intervention (ADOMIND) on adolescents’ depressive symptoms: a pilot study. L’Année psychologique. 2020;120(3):233. | Incorrect study design |
| Tran T, Huong Thanh Nguyen, Shochet IM, Nguyen N, La N, Wurfl A, et al. School-based universal mental health promotion intervention for adolescents in Vietnam: two-arm, parallel, controlled trial. Global mental health. 2023 Oct 23;1–35. | Incorrect study design |
| Tunariu AD, Tribe R, Frings D, Albery IP. The iNEAR programme: an existential positive psychology intervention for resilience and emotional wellbeing. International Review of Psychiatry. 2017 Jul 4;29(4):362–72. | Incorrect study design |
| Uhlig et al. “Being a bully isn’t very cool...”: rap & Sing Music Therapy for enhanced emotional self-regulation in an adolescent school setting-A randomized controlled trial. Psychology of music 2018;46(4):568â587. 2018. | Ineligible population |
| Eugénie Vaillant-Coindard, Gaëtan Briet, Lespiau F, Béatrice Gisclard, Charbonnier E. Effects of three prophylactic interventions on French middle-schoolers’ mental health: protocol for a randomized controlled trial. BMC psychology. 2024 Apr 13;12(1). | Incorrect study design |
| Venturo-Conerly KE, Osborn TL, Alemu R, Roe E, Rodriguez M, Gan J, et al. Single-session interventions for adolescent anxiety and depression symptoms in Kenya: A cluster-randomized controlled trial. Behaviour Research and Therapy. 2022 Apr;151:104040. | Incorrect intervention |
| Essi Viding, Lloyd A, Law R, Martin P, Lucas L, Tom Chin-Han Wu, et al. Trial protocol for the Building Resilience through Socio-Emotional Training (ReSET) programme: a cluster randomised controlled trial of a new transdiagnostic preventative intervention for adolescents. Trials. 2024 Feb 23;25(1). | Incorrect study design |
| Walker E, Corlett H, Hardacre C, Soulsby E, Frank K, Ling J, et al. P101 Feasibility of testing effectiveness of an interactive film to improve wellbeing in young people at school settings in the North of England. SSM Annual Scientific Meeting [Internet]. 2023 Aug 1 [cited 2025 Jan 23];A99.2-A100. | Incorrect study design |
| Wang FL, Feldman JS, Lemery-Chalfant K, Wilson MN, Shaw DS. Family-based prevention of adolescents’ co-occurring internalizing/externalizing problems through early childhood parent factors. Journal of consulting and clinical psychology [Internet]. 2019 Nov 1;87(11):1056–67. | Ineligible population |
| Wasil et al. Harnessing single-session interventions to improve adolescent mental health and well-being in India: development, adaptation, and pilot testing of online single-session interventions in Indian secondary schools. Asian journal of psychiatry 2020;50():101980. 2020. | Incorrect outcomes |
| Yaghoobi A, Moghadam BN. The effect of positive psychology intervention on the psychological well-being of adolescents. Iranian Journal of Psychiatry and Clinical Psychology 2019;25(1):14-25. 2019. | Incorrect study design |
| Yoon S, An S, Noh DH, Tuan LT, Lee J. Effects of health education on adolescents’ non-cognitive skills, life satisfaction and aspirations, and health-related quality of life: A cluster-randomized controlled trial in Vietnam. Stark L, editor. PLOS ONE. 2021 Dec 1;16(12):e0259000. | Incorrect intervention |
| Garvey W, Schembri R, Oberklaid F, Hiscock H. Feasibility, acceptability and outcomes of a health education intervention for children with emotional and behaviour difficulties: a pilot cluster randomised trial. Archives of Disease in Childhood. 2025 Jun 10;110(11):905–11. | Ineligible population |
| Rodgers NH, Zhang Y, Combiths P, Walker EA. Embedding Social–Emotional Learning in Elementary School–Based Speech-Language Therapy: A Pilot Randomized Controlled Trial. Language, Speech, and Hearing Services in Schools. 2025 Aug 8;1–19. | Ineligible population |
| Bradshaw CP, McDaniel H, Pas ET, Debnam KJ, Bottiani JH, Powell N, et al. Randomized controlled trial of the early adolescent coping power program: Effects on emotional and behavioral problems in middle schoolers. Journal of School Psychology. 2025 Jun;110:101437. | Incorrect intervention |
| Huang W, Wu P, Li J, Zhou Y, Xiong Z, Su P, et al. Effectiveness of a universal resilience-focused intervention for children in the school setting: A randomized controlled trial. Journal of Affective Disorders. 2025 Jan;368:695–703. | Ineligible population |
| Jong FC, Walker E, Corlett H, C. Hardarce, Soulsby E, Arnott B, et al. A cluster randomised feasibility trial assessing an interactive film intervention to improve wellbeing of young people in school settings in the North of England. Pilot and Feasibility Studies. 2025 Sep 2;11(1) | Incorrect study design |
| Cheung A. A Gamified Instrumental Musical Training to Enhance Resilience of Underprivileged School-aged Children at Risk of Mental Health Problems: A Randomised Controlled Trial. https://trial.medpath.com/clinical-trial/219349632ca9b4c0/nct07165925-gamified-musical-training-resilience-children-mental-health. | Incorrect intervention |
| Söderqvist F. Evaluating the Efficacy and Cost-effectiveness of the School-based Solution-focused Intervention for Mental Health (SIM) in First Year of Upper Secondary School Students. https://clinicaltrials.gov/study/NCT07111676 | Incorrect intervention |
| R Grasser L. School-based Practices in Arts and Resilience for Kids (SPARK Study). https://clinicaltrials.gov/study/NCT06945497 | Incorrect intervention |
| Yinyin Z. Enhancing Stress Resilience and Coping Skills Among Adolescents in Rural China Through School-Based Psychological Interventions: A Cluster Randomized Controlled Trial. https://clinicaltrials.gov/study/NCT07115186. | Incorrect intervention |
| Ricci Cabello I. Effects of a Multifaceted School-Based Intervention to Protect Mental Health and Prevent Suicidal Behaviour in Adolescents in Spain. https://clinicaltrials.gov/study/NCT06996054 | Incorrect intervention |
| Indonesia Endowment Fund for Education (LPDP), Republic of Indonesia  Effectiveness of Integrated Network for Student Psychosocial Intervention, Resilience, and Education (INSPIRE) on Mental Health Outcomes Among Indonesian Adolescents in Bandung (INSPIRE). https://clinicaltrials.gov/study/NCT06942637 | Incorrect intervention |
| X Yu N. Jockey Club Support Project to Enhance Emotion Regulation Skills and Resilience of Cross-Boundary Families: A Randomized Controlled Trial. https://clinicaltrials.gov/study/NCT07136064 | Incorrect intervention |
| Jong FC, Walker E, Corlett H, C. Hardarce, Soulsby E, Arnott B, et al. A cluster randomised feasibility trial assessing an interactive film intervention to improve wellbeing of young people in school settings in the North of England. Pilot and Feasibility Studies. 2025 Sep 2;11(1). | Incorrect study design |
| Morrell N. Pathways 2 Success: A Study of School-Based Prevention Programs Supporting Positive Adolescent Development. https://clinicaltrials.gov/study/NCT06831123 | Incorrect intervention |
| The Catholic University of America. Developing Resilience Skills and Social Competence in Youth in a School Setting. https://clinicaltrials.gov/study/NCT06578026 | Ineligible population |
| Mental Health Centre Copenhagen, Bispebjerg and Frederiksberg Hospital. Youth Aware of Mental Health (the YAM-project) - a School-based Program for Mental Health Promotion and Well-being in 9th Grade Students: a Cluster-randomized, Feasibility Trial. https://clinicaltrials.gov/study/NCT06549764. | Incorrect intervention |
| F Andrade B. Feasibility of a Social-emotional Learning Curriculum in an After-school Program for Youth. https://clinicaltrials.gov/study/NCT06619639 | Ineligible population |
| Malcolm-Smith S. Impact of Preventive Mental Health Programme on Social/Emotional Functioning and Resilience in Children in South Africa. https://clinicaltrials.gov/study/NCT06445777 | Incorrect intervention |
| Short-Term & Long-Term Benefits for Enhancing Educator & Student Prosocial Behavior, Well-being, & Resilience Through Mindfulness-Based Social and Emotional Learning Programs in Schools. Clinicaltrial.be. 2026 Available from: <https://clinicaltrial.be/nl/details/421513>? only_active=0&only_eligible=0&only_recruiting=0&per_page=100 | Incorrect study design |
| A Graham-Bermann S. Improving Mental Health in School-age Children Through the Kids' Empowerment Program (KEP) (KEP). https://clinicaltrials.gov/study/NCT06354907 | Incorrect study design |
| Vicary E, Brierley H, Wilkinson J, Adesina M, Bee P, Lovell K, et al. Bridging the gap in mental health literacy: co-adapting and feasibility testing a digital intervention to improve mental health literacy amongst young people aged 12–14 in the UK. Frontiers in Psychiatry. 2025 Jul 21;16. | Incorrect study design |
| Ünlü S, Uzun K, Arslan G. Mindfulness-Based Intervention in Schools: Addressing Social Media Burnout and Enhancing Well-Being in Adolescents. Children. 2025 Jun 23;12(7):826. | Incorrect intervention |
| Liu B, You J, Fan Y, Xia Y, Wang H, Zhang X, et al. Effect of team-building sports games on the resilience of Chinese rural children: evidence from Nanxian county, Hunan province. Frontiers in Pediatrics [Internet]. 2025 Mar 5;13. Available from: https://pmc.ncbi.nlm.nih.gov/articles/PMC11919669/pdf/fped-13-1552597.pdf | Incorrect outcomes |
| Li J, Hesketh T. A school-based intervention programme to prevent anxiety and depression among Chinese children during the COVID-19 pandemic. Child and Adolescent Psychiatry and Mental Health. 2024 Jun 17;18(1). | Ineligible population |
| Laurenzi CA, du Toit S, Mawoyo T, Luitel NP, Jordans MJD, Pradhan I, et al. Development of a school-based programme for mental health promotion and prevention among adolescents in Nepal and South Africa. SSM - Mental Health [Internet]. 2024 Jun 1 [cited 2024 Feb 6];5:100289. Available from: https://www.sciencedirect.com/science/article/pii/S2666560323001044 | Incorrect outcomes |
| Yang X, Zheng H. Intervention effect of CBT-based poetry therapy on promoting positive psychology: randomized controlled trials on Chinese adolescents. Current Psychology. 2025 May 14; | Incorrect outcomes |
| Lloyd A, Law R, Midgley N, Lucas L, Wu T (Chin-Han), Viding E, et al. Co-producing an interdisciplinary, preventative mental health intervention: development of the building resilience through socioemotional training (ReSET) programme. Advances in Mental Health. 2025 Sep 17;1–17. | Incorrect outcomes |
| Tang ACY, Lee RLT, Chan ACK, Kwan RYC, Lee PH. Mindfulness-based cognitive program as a potential intervention for managing smartphone addiction and resilience in adolescents: a pilot evaluation. Health Psychology and Behavioral Medicine. 2025 Jun 16;13(1). | Incorrect study design |
| Moran MJ, Aichele S, Shomaker LB, Lucas-Thompson RG, Heberlein E, Chandrasekhar JL, et al. Supporting Youth Mental Health Through a Health Coaching Intervention with a Mindfulness Component: A Pilot Randomized Controlled Trial During COVID-19. Child & youth care forum. 2023 Aug 10; | Ineligible population |
| Dadematthews A, Pangelinan MM, Bowen AE, Simon SL, Chandrasekhar JL, Kaar JL. The Effects of a Universal, School‐Based Mental Health Literacy Program to Improve Resilience and Self‐Efficacy Among 5–6th Grade Students From Three, Title 1 School in Rural Alabama: Advocates 4 All Youth. Psychology in the Schools; 2025. | Incorrect intervention |
| Rice KJ, Chen J, Kemps E, Roberts RM, Edwards S, Johnstone K. Two universal school-based prevention programs for depression and anxiety: 24-Month follow-up of an RCT. Journal of Behavior Therapy and Experimental Psychiatry [Internet]. 2024 Dec;85:101985. https://www.sciencedirect.com/science/article/pii/S0005791624000442 | Incorrect intervention |
| Srinivasan V, San Sebastián M, Rana S, Bhatt P, Armstrong G, Deshpande S, et al. Effectiveness of a resilience, gender equity and mental health group intervention for young people living in informal urban communities in North India: a cluster randomized controlled trial. Global Health Action. 2025 Feb 3;18(1). | Incorrect intervention |
