# Supplementary material for: Effectiveness of resilience-based interventions to promote mental well-being among secondary school children: a systematic review
Source: Front Psychiatry. 2026 Mar 5;17:1642660. doi: 10.3389/fpsyt.2026.1642660 (PMC13000754; doi:10.3389/fpsyt.2026.1642660)
Supplement: Supplementary file 4 [file Table1.docx]

**Table S1.** Characteristics of included studies in the review

| **Study ID** | **Study Design** | **Country/ Region** | **Intervention name** | **Mode of delivery** | **Frequency + Duration** | **Intervention programme** | **Control** | **Age (Mean/range)** | **Number of participants (N): I/C** |
| --- | --- | --- | --- | --- | --- | --- | --- | --- | --- |
| Ahmed et al. (2023) | Cluster RCT | Bangladesh | School-Based Physical Activity Intervention | Group sessions delivered by the research team | 60-minute session, once a week for 12 weeks | Participation in a range of multicomponent sessions, including circuit training, outdoor sports, health education, and infographics. | No intervention | 14.4 | 160/160 |
| Bogaert et al.  (2024) | Cluster RCT | Belgium | PET (Positive Events Training) | Group sessions delivered by trained researchers | Four in-class sessions, 50 min/week for 4 consecutive  weeks | Combination of in-class training to enhance positive autobiographical memory (AM) and episodic  future thinking skills to promote positive emotions and resilience. Worksheets were provided to support and encourage home practice. | Creative writing training | 12-16 | 95/93 |
| Burckhardt et al. (2016) | Cluster RCT | Australia | Strong Minds | Group sessions delivered by the researcher | Two sessions of 30 minutes per week for 8 weeks | Integration of Positive Psychology (PP) and Acceptance and Commitment Therapy (ACT) principles. Key PP components included gratitude, mindfulness, optimism, character strengths, and hope. ACT components focused on emotion acceptance, observer-self, committed action, and present moment awareness. | Pastoral care | 15-18 | 139/128 |
| Burckhardt et al. (2015) | RCT | Australia | The Bite Back | Online sessions managed by the website staff and teachers | 6 hours in total over a period of 4-6 weeks | Variety of online activities like gratitude entries, mindfulness meditations, and photography exercises to help youth apply positive psychology. It also provided information on nine positive psychology domains with suggestions for engaging with them outside the site. | Non-psychology entertainment websites | 14.7 | 313/259 |
| Buyukoksu et al. (2025) | RCT | Turkey | ACTi, “Promotion Resilience” | Group sessions delivered by teachers | One weekly session of 80-90 minutes for 6 weeks | The intervention consisted of a structured resilience programme based on positive psychology principles. Sessions focused on developing coping skills and emotional regulation, as well as raising awareness of personal strengths, through group discussions, guided activities and reflective exercises. | Education as usual | 12-15 | 36/36 |
| Chisholm et al. (2016) | Cluster RCT | United Kingdom | Contact + Education | Group-based sessions delivered by mental health staff | One day session of 5 hours | Mental health education lessons and a contact session where a 'Contact Volunteer' with lived experience of mental illness engaged with the class. The volunteer's mental health history was disclosed midway through the day, allowing students to interact without preconceptions. In the 20-minute Contact Session, the volunteer shared their experiences and answered questions. | Education alone | 11-13 | 354/303 |
| Dray et al.  (2017) | Cluster RCT | Australia | Intervention strategies across the Health Promoting Schools domains (WHO, 1991) | Group sessions delivered by teachers and support officers | 9 hours of intervention sessions + 9 hours of additional activities at school | The intervention involved 16 strategies across three Health Promoting Schools domains, targeting internal (e.g. cooperation, empathy, problem-solving) and external resilience factors (e.g. school support, peer relationships). Schools delivered nine hours of resilience-focused content in and outside the classroom. | Usual school curriculum | 12-14 | 2,823/1,766 |
| Green et al.  (2022) | RCT | United States | SPARK (Speaking to the Potential, Ability and Resilience Inside Every Kid) | Group sessions delivered by trained facilitators | One weekly session of one hour for 13 weeks | The program aimed to enhance youth resilience through social-emotional learning, as well as enhancing academic success through role plays, group discussions, and demonstrations. Key components included communication, problem-solving, decision-making, and emotional regulation skills. | Regular school curriculum | 15.7 | 200/172 |
| Ho et al.  (2017) | RCT | China | Sports-Based Youth Development Program | Group-based delivered by certified sports coaches | 90 minute-session per week for 18 weeks | After-school sports mentorship program focusing on youth empowerment and life skills development through Positive Youth Development (PYD) principles, offering activities like basketball, volleyball, or kickboxing, with an emphasis on goal setting and resilience building. | Online health education game | 12.32 | 346/346 |
| Johnson and Wade (2021) | Cluster RCT | Australia | Mindfulness Program | Group sessions delivered by an experienced mindfulness practitioner | One session of 65-75 min per week for 8 weeks | Mindfulness program with sessions including mindfulness concepts, informal mindfulness exercises, guided meditations, and group discussions led by an instructor to share experiences. | Usual school curriculum | 13-16 | 237/239 |
| Juul et al. (2025) | Cluster RCT | Denmark | “.b” mindfulness programme | Group sessions delivered by school teachers | One weekly session of 40-60 minutes for 10 weeks | Standardised mindfulness curriculum delivered through structured classroom lessons focusing on attention regulation, awareness of thoughts and emotions, and adaptive responses to stress. Activities included guided mindfulness practices, short reflective exercises, and classroom discussions. | Regular school curriculum | 12.3 | 836/892 |
| Kenny et al.  (2020) | Cluster RCT | Ireland | CopeSmart App | Individual, self-administered | Self-paced, but students were encouraged to engage with the app daily for 4 weeks | Mental health mobile app encouraging self-management by monitoring emotions and utilizing positive coping strategies. The app enabled adolescents to rate their feelings of happiness, anger, sadness, stress, or worry on a scale from 1 to 10, and users could track their ratings over time. | Control group, not specified | 15-18 | 385/175 |
| Kuyken et al.  (2022) | Cluster RCT | United Kingdom | SBMT (School-based mindfulness training) | Group sessions delivered by school teachers | 10 lessons of 30-50 min each for one school term | Program imparting mindfulness skills that bolster young people’s resilience through a mix of psychoeducation, class discussions, and short mindfulness exercises. Adapted for young people with varying levels of mental health, from those experiencing issues to those thriving. | Standard social-emotional training | 12.2 | 4,232/4,144 |
| Lam and Seiden  (2020) | RCT | China | L2B (Learning to BREATHE) | Group sessions delivered by a clinical and school psychologist | 6 sessions of 70 min each, once a month | Each session included a lesson presentation, activities to reinforce the theme, and in-class mindfulness practices. Sessions started with a review of ground rules and exercises such as focusing on sounds or breath awareness. Students received home practice handouts and optional guided audio files for external practice. | Religion and social studies lessons | 11-15 | 45/51 |
| Leventhal et al. (2015) | RCT | India | RC (Girls First Resilience Curriculum) | Group sessions delivered by Master trainers | One hour per week for 23 weeks | Girls identify personal strengths to set goals and learn coping strategies, positive psychology techniques, and emotional intelligence. They use these skills for problem-solving, conflict resolution, and in final sessions, girls collaborate to design and implement projects aimed at fostering peace in their own lives or the lives of others. | Regular school curriculum | 13.01 | 1,832/900 |
| Lipsey et al.  (2024) | RCT | United States | Healthy Minds | Online, self-administered | Single session lasting 30-45 minutes | Brief programme designed to promote a growth mindset about personality and emotions. It provided psychoeducational content and reflective writing exercises. | Online health education programme | 13-18 | 224/233 |
| Liu et al.  (2022) | Cluster RCT | China | MBI (Mindfulness-based intervention) | Group sessions delivered by trained instructors | 10 lessons of 45 min each, once a week | The program included modules on mindfulness concepts, such as Recognizing Worry, Being Here Now, and Befriending the Difficult. Alongside group practice, participants were encouraged to practice daily and document the content, duration, and frequency of their practice in an exercise manual. | Regular school program | 15.79 | 92/97 |
| Maalouf et al.  (2020) | RCT | Lebanon | FRIENDS program | Group sessions delivered by trained mental health professionals | 10 sessions of 45-50 min each for 12 weeks | The sessions address various concepts related to resilience and mental health, such as understanding emotions, practicing empathy, learning relaxation techniques, challenging negative thoughts, solving problems, and developing and maintaining friendships. | Waitlist control | 11-13 | 145/135 |
| Moore et al.  (2021) | RCT | Australia | Martial arts training | Group sessions delivered by a psychologist and a taekwondo instructor | One session of 50-60 min per week for 10 weeks | Combination of facilitator-led group discussions and martial arts training. The intervention included a psychoeducation component, covering topics like respect, goal setting, self-esteem, resilience, bullying, self-care, and optimism. | Waitlist control | 12-14 | 142/141 |
| Moran et al.  (2023) | RCT | United States | HK (Healthy Kids) | One-on-one, delivered in-person or virtually by school trained coaches | 30-minute session per week for 6 weeks | The first session focus on building rapport and introducing resilience. In the next sessions, brief mindfulness exercises are conducted, followed by discussions on resilience-building strategies. A long-term goal (4-month) is set collaboratively, with weekly action steps reviewed and documented by the coach and participant. | Assessment-only | 11.6 | 173/88 |
| Osborn et al. (2020) | RCT | Kenya | Shamiri-Digital Single session | Online session delivered by the researcher | One session of 2 hours; 30 min for questionnaires and 90 min for the intervention | Incorporation of three core elements from research on single-session interventions: Growth-mindset interventions to promote the belief that personal traits can develop and improve, gratitude interventions to cultivate appreciation for positive aspects of life, and value-affirmation interventions to help participants identify and reflect on their core values. | Program comprising note-taking skills and effective study habits modules | 15.36 | 50/53 |
| Pannebakker et al. (2019) | Cluster RCT | Netherlands | Skills 4 Life | Group sessions delivered by school teachers | One weekly lesson of one hour for 17 weeks during the first year. The second year included nine lessons | Cognitive-behavioural intervention based on Rational-Emotive Therapy and Social Learning Theory. The initial lessons introduce students to core principles, focusing on self-awareness of thoughts, feelings, and behaviours, while encouraging alternative thinking and correcting irrational reasoning. These lessons also develop general skills like problem-solving, emotion regulation, and critical thinking. | Usual curriculum | 13-16 | 964/541 |
| Puolakanaho et al. (2019) | RCT | Finland | Web-based Program Based on Acceptance and Commitment Therapy | Online and face-to-face sessions delivered by psychology coaches | 5-week duration with over 90 exercises, most of which lasted 5-10 minutes each | Program designed to improve adolescents' psychological flexibility. It guides them to explore their interests, thoughts, emotions, and sensations, set goals, and adjust behaviours accordingly. It teaches acceptance, mindfulness, self-compassion, and adaptation skills for personal and social life. | Support from school for psychological related issues | 15.25 | 81/82 |
| Rodgers and Dunsmuir (2015) | RCT | Ireland | FRIENDS for Life | Group session delivered by the researcher | One session of 60 min per week for 10 weeks | The program consists of three main components based on CBT principles: (a) Learning/Behaviour, (b) Cognitive, (c) Physiological. The program helps children and adolescents to develop problem-solving plans and coping strategies, practice positive self-talk, and apply relaxation techniques. | No active intervention | 12-13 | 32/30 |
| Saasa et al.  (2025) | Cluster RCT | Zambia | My FRIENDS Youth Program | Group sessions delivered by trained mental health facilitators | Two sessions of 90 minutes per week for 6 weeks | The programme focused on developing coping skills, emotion regulation, cognitive restructuring, and problem-solving through structured sessions. Activities included psychoeducation, group discussions, skills practice, goal setting, and homework tasks. | School activities as usual | 12.4 | 44/31 |
| Saelid et al.  (2022) | Cluster RCT | Norway | MindPower | Group sessions delivered by school teachers | Sessions of 1.5 hours per week for 8 weeks | Cognitive behavioural intervention aimed at helping adolescents understand mental health and enhance their ability to manage daily life stressors, strengthening their positive mental health and well-being. Topics covered include brain development, thinking patterns, and coping strategies. | Waitlist control | 15-16 | 540/393 |
| Santiago et al.  (2025) | Cluster RCT | United States | STRONG | Group sessions delivered by trained external facilitators | One weekly session of 60 minutes for 8 weeks | The programme combined cognitive-behavioural strategies, emotion regulation, and social-emotional skill development to support students’ mental health and wellbeing. Sessions included structured psychoeducation, group discussions, skills practice, and interactive activities. | Usual school activities | 11-14 | 32/32 |
| Seely et al.  (2023) | Cluster RCT | Germany | MHP-led prevention program | Group sessions delivered by a trained coach in CBT | One weekly session of 90 min for 10 weeks | The first sessions use self-management therapy to build student motivation through personal goal setting. The following sessions focus on understanding the connection between thoughts, emotions, and behaviours, teaching students to identify and challenge unrealistic or unhelpful thoughts. The final sessions focus on enhancing social skills through stories of coping role models. | Standard school curriculum | 14 | 213/226 |
| Suranata et al. (2020) | RCT | Indonesia | Internet-based Cognitive behavioural counselling | Individual, delivered by school counsellors | Unlimited | Cognitive-behavioural counselling aimed at improving students' emotional and behavioural health through cognitive restructuring (self-talk analysis, ABC training) and behavioural strategies (social skills, relaxation, problem-solving, and assertiveness training). | Waitlist control | 12 | 30/30 |
| Tak et al.  (2015) | Cluster RCT | Netherlands | OVK (Op Volle Kracht) | Group sessions delivered by psychologists | 16 lessons lasting 50 min each + 2-hour booster session at 12 months follow-up | The program's first eight lessons cover CBT principles, while the subsequent eight focus on coping strategies, decision-making, social skills, and problem-solving techniques. Adolescents use a workbook for assignments and to practice skills through role-playing, discussions, and tasks, with homework to help internalize and apply the skills learned. | School as usual | 13.95 | 655/735 |
| Tokolahi et al. (2018) | Cluster RCT | New Zealand | Kia Piki te Hauora | Group sessions delivered by an occupational therapist | One session of an hour per week for 8 weeks | The occupational therapy intervention promotes mental health by engaging students in activities that link actions with their feelings/thoughts, support self-concept, and develop strategies for managing difficult emotions. It also helps establish healthy routines and behaviours to enhance self-esteem and active participation. | Waitlist control | 11-13 | 69/71 |
| Volanen et al. (2020) | Cluster RCT | Finland | .b (Stop and Breathe/Be | Mix of group and individual sessions delivered by trained mindfulness facilitators | Nine weekly 45-min sessions and short home practice (recommended amount of 5-6 times per week) | The program consists of 9 interactive classroom lessons designed to develop skills for enhancing happiness and well-being, techniques for relaxation, stress management, focus, and fostering better relationships, while also helping students understand their own minds and the factor contributing to a healthy life. | Standardized relaxation  program | 12-15 | 1,646/1,488 |
| Zhou et al.  (2023) | Cluster RCT | China | Coping Camp app | Online, self-administered | One weekly lesson of 25–45 minutes for 11 weeks | The intervention is modelled on Stress Inoculation Training, a form of Cognitive Behavioural Therapy designed to reduce stress. It includes 11 sessions focusing on somatic skills (mindfulness), cognitive restructuring skills, behavioural skills (goal setting, time management), and interpersonal skills | Regular moral education classes | 16.76 | 275/265 |
| Zou & Liu (2025) | RCT | China | Physical activity programme | Mix of group and individual sessions delivered by trained instructors | Three sessions of 60 minutes per week for 12 weeks | Sessions combined team-based sports, individual fitness exercises, breathing and relaxation activities, and short group reflections. The programme aimed to strengthen coping skills, perseverance, and emotional regulation through structured physical activity and supportive peer interaction. | Non-physical activity group sessions | 12-17 | 100/100 |
