# Supplementary material for: Effectiveness of resilience-based interventions to promote mental well-being among secondary school children: a systematic review
Source: Front Psychiatry. 2026 Mar 5;17:1642660. doi: 10.3389/fpsyt.2026.1642660 (PMC13000754; doi:10.3389/fpsyt.2026.1642660)
Supplement: Supplementary file 5 [file Table2.docx]

**Table S2.** Short-term outcome effects (<6 months)

| **Study ID** | **Length of follow-up** | **Outcome Domain** | **Outcome Instrument** | **Effect size** | **95% CI** | **p-value** | **Summary of findings** |
| --- | --- | --- | --- | --- | --- | --- | --- |
| Ahmed et al. (2023) | Post- intervention at 12 weeks | Depressive symptoms | CESD- 10 item | -4.60 | -5.76, -3.46 | <0.001 | The effect size and 95% CI suggest a statistically significant, large reduction in depressive symptoms in favour of the intervention. |
| Bogaert et al. (2024) | 8 weeks | Resilience  Anxiety symptoms  Depressive symptoms | CD-RISC  DASS-21  DASS-21 | 0.005  0.002  0.008 | N/A | N/A | The results of the study showed minimal effects across the different outcome domains. The effect sizes for resilience, anxiety symptoms, and depressive symptoms were very small. |
| Burckhardt et al. (2016) | Post- intervention at 12 weeks | Anxiety symptoms  Depressive symptoms | DASS-21  DASS-21 | 0.01  0.53 | N/A | 0.35  0.04 | Results showed that participants in the intervention group experienced significant reductions in depression scores compared to the control group, with a medium effect size. For anxiety symptoms, no significant effect was found. |
| Burckhardt et al. (2015) | Post- intervention at 6 weeks | Depressive symptoms  Anxiety symptoms | DASS-21  DASS-21 | N/A | N/A | 0.52  0.60 | The intervention had no significant effect on either depression or anxiety outcomes when compared to the control group, regardless of baseline symptom severity or the level of participant engagement. |
| Buyukoksu et al. (2025) | Immediately post-intervention | Resilience | CD-RISC | 0.492 | N/A | <0.01 | The intervention led to a statistically significant improvement in resilience, with higher post-intervention scores observed in the intervention group compared with baseline. |
| Chisholm et al. (2016) | 2 weeks | Resilience | 15-item version of the RS | 0.16 | -0.16, 0.48 | 0.3 | The results for resilience showed a small effect size. However, the 95% confidence interval and p-value indicate that this result was not statistically significant. |
| Green et al. (2022) | Post- intervention at 13 weeks | Resilience | RSCA | 0.78 | N/A | <0.0001 | The intervention yielded a positive impact on increasing resilience, which is supported by a large effect size and a p-value indicating a strong statistically significant result. |
| Ho et al. (2017) | 4 weeks | Resilience | CD-RISC | 0.19 | 0.03, 0.34 | 0.02 | The outcome for resilience showed a small but statistically significant effect size, favouring the intervention. |
| Johnson and Wade (2021) | 12 weeks | Anxiety symptoms  Depressive symptoms | GAD-7  DASS-21 | -0.09  -0.04 | -0.27, 0.10  -0.23, 0.15 | N/A | The effect sizes for anxiety and depressive symptoms suggest that the intervention had no impact on reducing these measures after 3 months of follow-up. |
| Kenny et al. (2020) | 8-12 weeks | Emotional distress | DASS-21 | N/A | N/A | N/A | The results showed that there were no significant differences in DASS-21 scores between the intervention and control group, suggesting that the app did not have a significant effect on psychological distress. |
| Lam and Seiden (2020) | Post- intervention at 20 weeks | Internalizing problems | YSR | 0.04- 0.05 | N/A | 0.02 | The results yielded significant effects on internalizing problems in favour of the intervention, although the effect size was very small. |
| Leventhal et al. (2015) | Post- intervention at 20 weeks | Anxiety symptoms  Depressive symptoms  Emotional Resilience | GAD-7  PHQ-9  CD-RISC | 0.15  N/A  0.46 | N/A | 0.025  >0.05  <0.01 | The intervention had a positive impact on emotional resilience, with a statistically significant effect size of 0.46. Anxiety scores increased more in the intervention group than in the control group, although the difference’s magnitude was small. No significant intervention effect was found for depression. |
| Lipsey et al.  (2024) | Post-intervention immediate post-test | Depressive symptoms | SMFQ | N/A | N/A | N/A | The results showed no significant differences in depressive symptoms between the Healthy Minds intervention and the control condition at post-intervention, indicating that the brief online growth mindset intervention did not have a significant short-term effect on depression. |
| Liu et al. (2022) | Post- intervention at 10 weeks | Psychological Resilience | RSCA* | 0.90 | 0.59, 1.21 | <0.01 | The intervention showed a large effect size on psychological resilience, indicating a significant increase in scores for the experimental group at post-test. |
| Maalouf et al. (2020) | Post- intervention at 12 weeks | Depressive symptoms  Generalized anxiety disorder | MFQ  SCARED | N/A | N/A | 0.039  >0.05 | The study suggested that individuals who were part of the intervention group were associated with a significant reduction in depressive symptoms over time, in comparison to the control group. No statistically significant effect was observed for the total SCARED score. |
| Moore et al. (2021) | 12 weeks | Total Resilience | CYRM | 0.11 | -0.03, 0.25 | N/A | The intervention yielded a non-significant effect on participants’ total resilience scores at follow-up. However, at post-intervention (10 weeks), there was a significant effect, which suggests that it was not sustained over time. |
| Moran et al. (2023) | Post- intervention at 6 weeks | Resilience  Depressive symptoms  Anxiety symptoms | CYRM  PROMIS  PROMIS | 0.02  0.23  0.20 | N/A | 0.18  0.12 | No significant differences were observed between the groups in the changes from baseline to post-intervention for depression symptoms, anxiety symptoms (p > 0.15), and resilience. |
| Osborn et al. (2020) | 2 weeks | Anxiety symptoms  Depressive symptoms | GAD-7  PHQ-8 | 0.29  0.50 | -0.20, 0.79  0.00, 1.6 | 0.280  0.028 | Adolescents in the intervention showed a moderate reduction in depressive symptoms from baseline to the 2-week follow-up compared to those in the control group. The observed reduction in anxiety symptoms among the intervention group did not reach statistical significance. |
| Puolakanaho et al. (2019) | Post- intervention at 5 weeks | Academic buoyancy | ABS | 0.27 | N/A | 0.013 | There was a statistically significant increase in academic buoyancy among all participants, although the effect size was small. |
| Rodgers and Dunsmuir (2015) | 16 weeks | Anxiety symptoms | SCAS | N/A | N/A | N/A | The results showed a significant interaction between group and time, indicating that the intervention group's anxiety scores reduced significantly between pre-intervention and post-intervention, while the control group did not. However, the study did not provide a specific effect size. |
| Saasa et al.  (2025) | 12 weeks | Anxiety symptoms  Depressive symptoms | GAD-7  PHQ-9 | -2.26  0.22 | N/A | <0.01  0.767 | The intervention was associated with a statistically significant reduction in anxiety symptoms, indicating a moderate magnitude of improvement. In contrast, no significant effect was observed for depressive symptoms. |
| Saelid et al. (2022) | 20 weeks | Anxiety symptoms  Depressive symptoms | SCL-8  RADS-2:SF | N/A | N/A | N/A | The overall analysis showed no significant changes in symptom scores for either anxiety or depression. |
| Santiago et al.  (2025) | 12 weeks | Depression/Anxiety  Resilience  Externalising problems | RCADS  CD-RISC  SDQ | -6.03  3.18  -1.28 | N/A | <0.05  <0.01  <0.01 | The intervention resulted in a significant reduction in combined anxiety and depressive symptoms, and externalising problems. No significant improvement in resilience was observed immediately post-intervention. However, resilience increased significantly at delayed follow-up, suggesting potential longer-term benefits. |
| Suranata et al. (2020) | 5 weeks | Resilience | RYDM | 0.862 | N/A | <0.05 | The intervention had a significant impact on resilience at the post-test and five-week follow-up, demonstrated by the large effect size on resilience that was sustained over time. |
| Tokolahi et al. (2018) | Post- intervention at 8-9 weeks | Anxiety symptoms  Depressive symptoms | MASC-10  CDI- 2^nd^ edition | 3.0  0.2 | -0.4, 6.3  N/A | 0.082  0.880 | The intervention did not have significant effects on anxiety or depressive symptoms. |
| Zhou et al. (2023) | 19 weeks | Anxiety symptoms  Depressive symptoms | DASS-21  DASS-21 | -0.13  0.11 | N/A | 0.01  0.05 | At the 19-week follow-up, the effects of the intervention were significant for anxiety symptoms. However, there was no significant impact for depressive symptoms. |
| Zou & Liu (2025) | Immediately post-intervention | Resilience | CD-RISC-25 | 2.17 | N/A | <0.001 | The intervention led to a statistically significant improvement in resilience, with the intervention group showing a large increase in resilience from pre- to post-intervention compared with the control group. |

CESD, Center for Epidemiologic Studies Depression Scale; CD-RISC, Connor-Davidson Resilience Scale; DASS-21, Depression Anxiety and Stress Scale; RS, Resilience Scale; RSCA, Resiliency Scales for Children and Adolescents; GAD-7, 7-item Generalised Anxiety Disorder Scale; YSR, Youth Self-Report; PHQ-9, Patient Health Questionnaire-9; RSCA*, Resilience Scale for Chinese Adolescents; MFQ, Mood and Feelings Questionnaire; SCARED, Scale for Childhood Anxiety and Related Disorders; CYRM, Child and Youth Resilience Measure; PROMIS, Patient Reported Outcome Measurement Information System, Pediatric Anxiety and Depressive Symptoms Scales; PHQ-8, Patient Health Questionnaire-8; ABS, Academic Buoyancy Scale; SCAS, Spence Children’s Anxiety Scale; SCL-8, Symptom Check Measure Assessment List-8; RCADS, Revised Children’s Anxiety and Depression Scale; SDQ, Strengths and Difficulties Questionnaire; RADS-2:SF, Reynolds Adolescent Depression Scale; RYDM, Resilience Youth Development Module- Indonesian version of psychological subscale; CDI, Children’s Depression Inventory; MASC-10, Multidimensional Anxiety Scale for Children- Short form.
