# Supplementary material for: Effectiveness of resilience-based interventions to promote mental well-being among secondary school children: a systematic review
Source: Front Psychiatry. 2026 Mar 5;17:1642660. doi: 10.3389/fpsyt.2026.1642660 (PMC13000754; doi:10.3389/fpsyt.2026.1642660)
Supplement: Supplementary file 6 [file Table3.docx]

**Table S3.** Long-term outcome effects (>6 months)

| **Study ID** | **Length of follow-up** | **Outcome Domain** | **Outcome Instrument** | **Effect size** | **95% CI** | **p-value** | **Summary of findings** |
| --- | --- | --- | --- | --- | --- | --- | --- |
| Dray et al. (2017) | 3 years | Resilience  Internalising problems  Externalising problems  General psychological distress | CHKS  SDQ subscale  SDQ subscale  Total SDQ | -0.01  0.05  0.43  0.47 | -0.07, 0.06  -0.54, 0.63  0.04, 0.83  -0.41, 1.35 | 0.81  0.87  0.02  0.27 | The intervention was not found to have a statistically significant impact on resilience, internalising problems, or general psychological distress. However, there was a significant difference in externalising problems favouring the control group, although the effect size was small. |
| Juul et al. (2025) | 8 months | Resilience | BRS | -0.04  -0.03 | N/A  N/A | 0.303  0.459 | The intervention did not produce a significant effect on resilience. Across the total student population, small and non-significant between-group differences were observed at 5- and 8-month follow-up. |
| Kuyken et al. (2022) | 1 year | Anxiety symptoms  Depressive symptoms  Conduct problems | RCADS  CESD  SDQ | 0.4  0.1  -0.1 | -1.0, 1.9  -0.6, 0.7  -0.2, 0.1 | 0.56  0.86  0.30 | At 1-year follow-up, there were no differences observed for any of the outcomes. The effect sizes and their 95% CI, indicated that important effects could be ruled out. |
| Pannebakker et al. (2019) | 20 months | Depressive symptoms | BDI, 22 items | -0.26 | N/A | 0.02 | The intervention suggested that depressive symptoms decreased significantly from baseline to the 20-month follow-up in the intervention group compared to the control group, though the effect size was small. |
| Seely et al. (2023) | 1 year | Emotional symptoms  Conduct problems | SDQ- subscale  SDQ- subscale | 0.30  0.11 | N/A  N/A | 0.029  0.435 | The intervention group showed a small significant effect on emotional symptoms compared to the control group. For conduct problems, the effect was also small but did not reach statistical significance. |
| Tak et al. (2015) | 1 year | Depressive symptoms | CDI | 0.02 | -0.08, 0.13 | 0.829 | The intervention did not prevent depressive symptoms. Moreover, the prevalence of elevated depressive symptoms did not differ between groups at the 1-year follow-up. |
| Volanen et al. (2020) | 26 weeks | Resilience  Depressive symptoms | RS14  BDI | 0.01  -0.07 | N/A | N/A | The intervention did not have a significant impact on resilience and depressive symptoms at the 26-week follow-up. |

CHKS, Resilience and Youth Development Module of the California Healthy Kids Survey; SDQ, Strengths and Difficulties Questionnaire; BRS, Brief Resilience Scale; RCADS, Anxiety subscales from the Revised Child Anxiety and Depression Scale; CESD, Center for Epidemiologic Studies Depression Scale; BDI, Beck Depression Inventory; CDI, Children’s Depression Inventory; RS14, Resilience Scale.
